# Comprehensive Real-Time Analysis of the Yeast Volatilome

**DOI:** 10.1038/s41598-017-14554-y

**Published:** 2017-10-27

**Authors:** Alberto Tejero Rioseras, Diego Garcia Gomez, Birgitta E. Ebert, Lars M. Blank, Alfredo J. Ibáñez, Pablo M-L Sinues

**Affiliations:** 1 Department of Chemistry and Applied Biosciences, ETH Zurich, 8093 Zurich, Switzerland; 2 SEADM S.L., C\ José Lázaro Galdiano, 1, Madrid, 28036 Spain; 3 Department of Analytical Chemistry, University of Cordoba, Cordoba, Spain; 4 Institute of Applied Microbiology – iAMB, Aachen Biology and Biotechnology – ABBt, RWTH Aachen University, Worringerweg 1, Aachen, 52074 Germany; 5 Instituto de Ciencias Ómicas y Biotecnología Aplicada - Pontificia Universidad Católica del Perú (ICOBA-PUCP), Lima, Peru; 6 University Children’s Hospital Basel, Department of Biomedical Engineering, University of Basel, Basel, Switzerland

## Abstract

While yeast is one of the most studied organisms, its intricate biology remains to be fully mapped and understood. This is especially the case when it comes to capture rapid, *in vivo* fluctuations of metabolite levels. Secondary electrospray ionization-high resolution mass spectrometry SESI-HRMS is introduced here as a sensitive and noninvasive analytical technique for online monitoring of microbial metabolic activity. The power of this technique is exemplarily shown for baker’s yeast fermentation, for which the time-resolved abundance of about 300 metabolites is demonstrated. The results suggest that a large number of metabolites produced by yeast from glucose neither are reported in the literature nor are their biochemical origins deciphered. With the technique demonstrated here, researchers interested in distant disciplines such as yeast physiology and food quality will gain new insights into the biochemical capability of this simple eukaryote.

## Introduction

Metabolites are small molecules that provide a direct readout of organisms’ phenotypes and cellular activity^1^. During metabolic processes^2^, a subset of the metabolites, the so-called volatile organic compounds (VOCs) are released to the ambient at normal pressure and temperature. VOCs are organic compounds typically C_5_-C_20_^3^ with molecular weights up to 500 Da^4^, boiling point up to 250 °C^5^ and high vapor pressure. Many living organisms including humans, animals, plants, and even microorganisms, produce large varieties of VOCs^6^. The sum of all VOCs produced by an organism has been termed the volatilome^7–9^. While some VOCs have dedicated pathways, many are intermediates of anabolic pathways or are synthesized by moonlighting activities of enzymes^10^, hence reflect promiscuous enzymatic activities in the underlying metabolic network^11,12^. Although generally produced in small concentrations (parts-per-million to parts-per-trillion range)^13^, VOCs reflect the metabolic state of a cell and, thus, can be used to understand many biological processes such as, e.g., oxidative stress^14–16^. VOCs are also at the core of the flavor and fragrance manufacturing, an industry with a focus on plant-derived VOCs. Volatile metabolites are also of importance in the food industry as taste and smell is informing the consumer on the quality of a product. A volatile metabolite, industrially produced in large quantities, is alcohol (i.e., ethanol), synthesized by yeast from glucose during fermentation. Interestingly, since ethanol is neutral-flavored, the taste of fermented drinks originates from minor compounds, such as higher alcohols, aldehydes, esters, and acids. Most of these metabolites are volatile and intensely flavored. Indeed, monitoring VOCs (i.e., diacetyl, 2,3-pentanedione) abundance in beer is industrial standard, since it has a strong effect on the product quality^17^. The economic impact of monitoring such compounds is thus vast^18^.

Gas chromatography-mass spectrometry (GC-MS)-based methods have been the workhorse to analyze VOC profiles. The main limitation of such methods is that it requires sample manipulation, resulting in laborious procedures^19,20^. A more recently deployed approach is ion mobility spectrometry, which provides near real-time analysis, but its poor resolution compared to mass spectrometry compromises metabolite coverage and compound identification capabilities^21–24^. Real-time mass spectrometric methods used for on-line quantification of a handful of VOCs include proton transfer reaction (PTR)^13^ and selected ion flow tube (SIFT)^25^. A recent mass spectrometric method based on direct flow injection, has shown to monitor metabolite dynamics in the 15–30 second range and was used to gain insights into cellular responses to environmental changes^26^.

In line with such efforts to develop instrumentation capable of monitoring time-resolved metabolic information, we show here how secondary electrospray ionization (SESI)^27–39^ coupled to high resolution mass spectrometry (HRMS) captures on-line an unprecedented wealth of volatile analytes emitted *in vivo* by growing baker’s yeast (*Saccharomyces cerevisiae*).

## Results and Discussion

### Volatiles emitted by wild type Saccharomyces cerevisiae during growth in^13^C_6_-glucose

**Figure 1:**
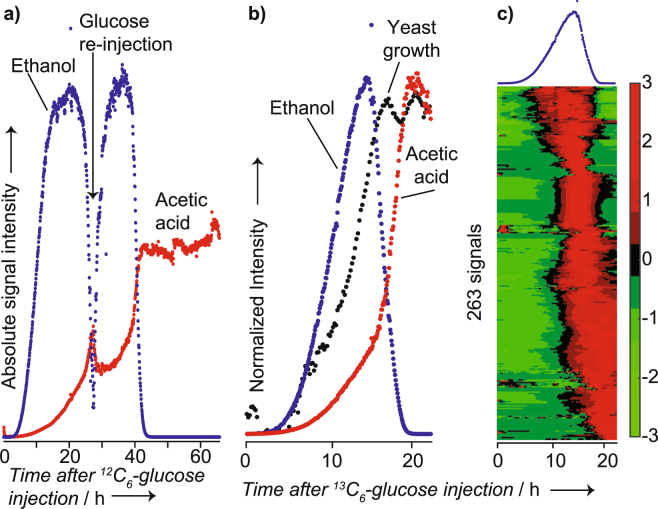
Real-time monitoring of volatile metabolites during yeast growth; (**a**) raw time profiles of ethanol (detected as the dimer) and acetic acid during growth on ^12^C_6_-glucose and re-injection of glucose after ~27 h; (**b**) normalized raw time profiles of ethanol (detected as the dimer), acetic acid and the image signal captured by a time-lapse camera during growth on ^13^C_6_-glucose; c) heatmap showing 263 time-dependent signals during yeast growth. All the signals in the heatmap were labeled with ^13^C. For reference, the ethanol signal is shown on the top.

Initially, to confirm that SESI-HRMS is indeed suitable to track growth-related metabolites in real-time, we grew the yeast in glucose minimal medium and monitored well-known end products^40^ like ethanol or acetic acid. As expected, we observed the production of ethanol despite full aeration because baker’s yeast is Crabtree positive. Figure 1a shows such an example, in which ethanol production kicked off shortly after the start of the growth experiment. The concentration increased exponentially to then level off, presumably concomitant with glucose depletion. Eventually, ethanol started to decrease again. Such a diauxic growth is expected for yeast fermentation on glucose. The depletion kinetic, however, seems too fast to be solely explained by metabolization. Evaporation of this volatile metabolite might have been enforced by the vigorous aeration (25 gas volume flow per unit of liquid volume per minute; vvm) of the bioreactor. When ethanol was almost completely depleted, we spiked glucose into the medium (~27 h; see Methods section for experimental details). The cells immediately re-started glucose consumption as evidenced by the instantaneous production of ethanol and acetic acid.

One of the advantages of SESI coupled to high-resolution mass spectrometry (HRMS) is that it can detect a wide range of analytes, including species of low vapor pressure at concentrations in the low parts-per-trillion range^41^. Indeed, along with the well-known volatile metabolites ethanol and acetic acid, numerous other volatile metabolites were detected during yeast growth. Thus, we replicated the experiment independently; however, we administered glucose labelled with ^13^C in all six carbon atoms. ^13^C label incorporation into metabolites synthesized from glucose allowed differentiating analytes of biological origin from environmental contaminants or media components. Figure 1b shows the resulting time profiles of ethanol, acetic acid, and yeast biomass estimated from image grayscale levels in a time-lapse video. The observed correlation between increase of ethanol and production of biomass confirmed that the analytical setup allowed capturing biological events soundly. ^13^C-label incorporation confirmed that 263 signals observed in the previous experiment originated from the yeast’s metabolism. In addition, we conducted a negative control experiment whereby a bioreactor was run under the very same conditions but without inoculation with yeast (monitored for ~17 hours). As expected, neither ethanol nor acetic acid were produced (Figure S1).

The time-resolved dataset of the yeast fermentation enabled a comprehensive overview of the volatilome dynamics (Fig. 1c). The heatmap is ordered by a cluster analysis, easing the visualization of the different time-profiles. Note that these metabolites were captured simultaneously and in real-time without any sample manipulation. Table S1 lists the 263 signals detected in both experiments (i.e., labeled and non-labeled glucose) as they appear in the heatmap of Fig. 1c. Interestingly, homologous series of compounds tend to cluster together, suggesting similar kinetics for closely related metabolites. For example, one cluster comprised the homologous series C_6_H_10_, C_7_H_12_, C_8_H_14_ and C_9_H_16_. This bouquet of volatile metabolites remains to be annotated, but the chemical formula suggests that these compounds could well be alkadienes. Several olefinic compounds have been reported for diverse organisms^42–46^. 1,3-octadiene (C_8_H_14_), for example, is produced by several fungi and predicted to be a degradation product of linoleic acid, but the responsible enzymes and encoding genes are yet to be identified^47^. None of the potential alkadienes detected here were previously identified in the headspace of yeast fermentations.

### Volatiles emitted by wild type and mutants of *Saccharomyces cerevisiae* during growth in ^13^C_1_-glucose

**Figure 2:**
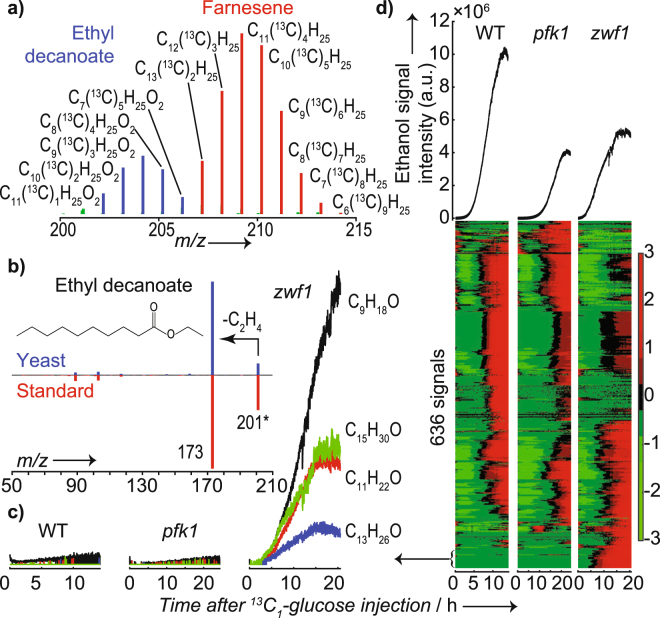
Growth of WT and mutants on ^13^C_1_-glucose led to complex mass spectra that revealed a unique *in vivo* metabolic response; (**a**) Centroided mass spectrum of the region *m/z* 200–215 during the stationary phase of WT yeast growth upon injection of ^13^C_1_-glucose into the system. Two isotopic envelopes for C_12_H_25_O_2_ and C_15_H_25_ were clearly resolved. The incorporation of ^13^C into ethyl decanoate and farnesene reflects the metabolism of the yeast; (**b**) Fragmentation (SESI MS/MS) spectrum produced using *m/z* 201 as the precursor ion from a yeast sample (top) and ethyl decanoate standard (bottom); (**c**) Set of odd-numbered carbon molecules built up during growth of the *zwf1* mutant but absent in WT and *pfk1*; (**d**) Heatmap for the 636 signals detected for the three strains. For reference, ethanol profiles are shown on the top.

To demonstrate that the detected series of hydrocarbons were produced by the baker’s yeast and not by a biological contamination of the bioreactor, we repeated the measurements. Specifically, we performed a comparison of two baker’s yeast mutants (i) devoid of the oxidative pentose phosphate pathway (*zwf1*), which is associated with oxidative stress response or (ii) limited in glycolysis (*pfk1*); and compared them with a wild type (WT) strain. To discriminate *in vivo* pathway usage, we fed WT, *zwf1*, and *pfk1* with ^13^C_1_-glucose. Figure 2a shows a zoom of the *m/z* range 200–215 of a typical mass spectrum acquired during such measurements for WT during the stationary phase. It shows two distinct bell-shaped isotopic distributions. The first one assigned to a series of C_x_(^13^C)_y_H_25_O_2_ with x ranging 11 to 7 and y 1 to 5 (i.e., molecular formula C_12_H_25_O_2_). Similarly, another more abundant distribution of C_15_H_25_ (^13^C ranging from 2 to 9) was clearly observed. Apart from high resolution (~ 30,000) enabling the discrimination of complete ^13^C isotopic envelopes, MS/MS capability is just another advantage of our proposed technique over other common on-line technologies used to monitor volatiles in industrial processes^48^. Thus, further insights in this respect were gained by identifying some of the detected compounds. For example, the two compounds in Fig. 2a were assigned to ethyl decanoate and farnesene based on the MS/MS spectrum (Figs 2b and S2). Ethyl esters have fruity and floral flavor and constitute important aroma compounds in wine or beer^49^. It is hypothesized that fatty acid ethyl esters are formed to prevent accumulation of medium chain fatty acid under anaerobic conditions during which fatty acid synthesis is inhibited and these fatty acids are prematurely released from the fatty acid synthase^50^. The sesquiterpene farnesene is synthesized from farnesyl diphosphate, an intermediate of the mevalonate pathway.

Figure 2c shows an example of some representative time profiles for a set of metabolites present in the *zwf1* mutant but absent in WT and *pfk1*. Notably, this set of metabolites is a homologous series of odd-numbered carbons ranging from C_7_ to C_15_. This observation was replicated in an independent experiment (Figure S3). We hypothesize that these compounds are methyl ketones, which can be derived from β-oxidation of fatty acids, but their identity remains to be fully elucidated^51^. Likewise, some analytes were characteristic for *pfk1* (see example in Figure S4).

Figure 2d (top) shows the time traces for ethanol. We found that the WT produced approximately twice as much ethanol as the mutants under the same experimental conditions. The same results were obtained in a replicate experiment (Figure S3). This is consistent with previous work, where similar, reduced glucose uptake and growth rate ―and as a consequence, a low ethanol formation rate― was found for the *zwf1* mutant^52^. As for Fig. 1c, we subjected the time traces for all detected signals of the three strains to a cluster analysis and visualized them in heatmaps. Figure 2d (bottom) shows the results, providing an overview of the differences in volatile profiles between the strains. While the general picture seems relatively similar for WT and *pfk1*, the *zwf1* mutant showed marked differences for some compounds. For example, at the bottom of the heatmap one can observe the cluster of molecules from Fig. 2b. Table S2 lists all the peaks detected in this experiment. Overall, this initial assessment illustrates the potential of SESI-HRMS to provide a comprehensive overview of VOC production by baker’s yeast in real-time.

Although the *pfk1* cells showed a different metabolic profile than WT and *zwf1* cells, our study showed an unexpected result. The *pfk1* strain was able to undergo glycolysis (apparent from lower than expected loss of ^13^C) after an extended time of lag-phase growth. It is not entirely clear to us, how the *pfk1* strain was able to accomplish this. However, *S*. *cerevisiae*’s phosphofructokinase is a heterooctameric enzyme with an alpha subunit encoded by PFK1 and a beta subunit encoded by PFK2. Thus, a single gene deletion mutant deficient in either of these genes might retain phosphofructokinase activity, which may explain the observed labeling pattern^53,54^.

**Figure 3:**
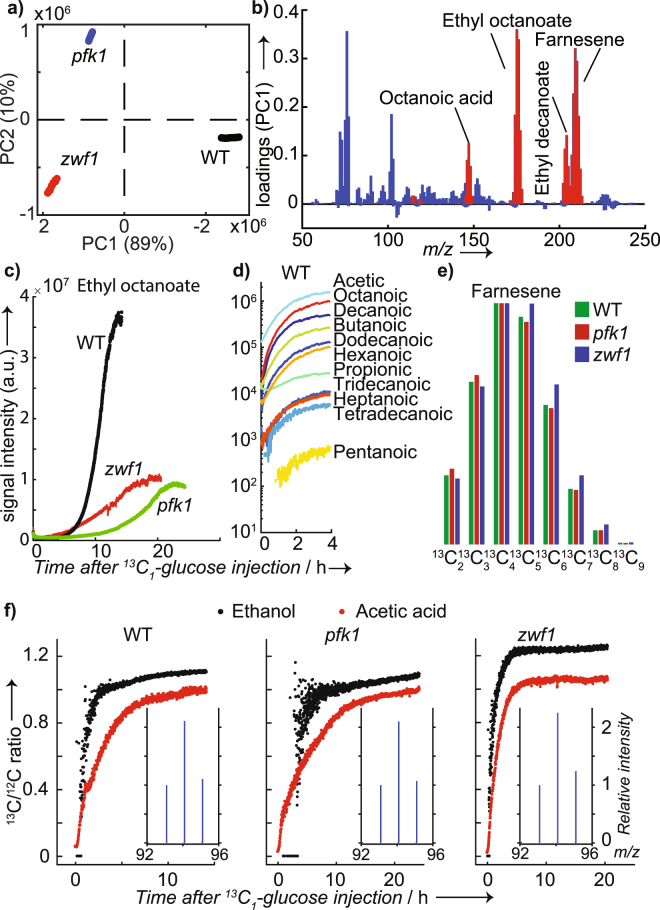
Distinct volatile metabolic profiles and production kinetics for yeast WT and mutants; (**a**) PCA score plot of average spectra in the exponential phase for WT and mutants suggests a clear distinction based on the volatile metabolic profile; (**b**) the corresponding loading plot for score 1 shows that esters, acids, and sesquiterpenes are major contributors to the separation between WT and mutants. Identified compounds are highlighted in red. Note that ethanol and acetic acid were excluded from the PCA analysis; (**c**) Kinetic profiles of the food-relevant metabolite ethyl octanoate for WT, *pfk1* and *zwf1* illustrates the potential to monitor industrial processes; (**d**) series of fatty acids detected in negative ion mode during WT growth in ^13^C_1_-glucose; (**e**) isotopic distribution for farnesene obtained during the stationary phase. As expected, it shows a greater accumulation of ^13^C for *zwf1*; (**f**) Time profiles of ^13^C/^12^C ratios for ethanol dimer (i.e., *m/z* 95/93; black) and acetic acid (red) for the three strains investigated. Note the different kinetic profiles. The average spectra during the stationary phase for the ethanol dimer is shown in the insets.

Another common approach to visualize multivariate data is principal component analysis (PCA). The score plot of the mass spectra during the exponential phase (Fig. 3a) clearly shows a separation between the three strains, confirming the existence of a distinct volatile profile for each strain. Please note that we have excluded ethanol and acetic acid from the analysis in order to show that even without these major end-products, the less abundant volatile compounds suggest a very distinct profile. As expected, a similar result was obtained when we included ethanol and acetic acid in the PCA matrix (Figure S5). To understand which metabolites contributed mostly to the separation shown in the score plot, Fig. 3b displays the loadings for the first PC, which separate WT from the mutants. Some other highly discriminating molecules were identified via MS/MS and by comparison with standards (Figure S2). The esters ethyl octanoate and ethyl decanoate were major contributors, along with farnesene and octanoic acid. Tables S3 and S4 list the major contributions for PC1 and PC2, respectively. When ethanol was included in the analysis, it was by far the major contributor to the separation shown in the score plot (Figure S5).

Some of the identified compounds are not only relevant biologically, but also industrially. For example, ethyl octanoate is a yeast-based food product with sour apple aroma^55^. Figure 3c shows the time traces for ethyl octanoate for the three strains investigated. It clearly shows that the levels produced by WT were about four times as much as that of the mutants, reinforcing the potential to monitor industrial processes. Interestingly, ethyl hexanoate showed a very similar trend (this and additional relevant examples are shown in Figure S6). Fatty acids such as hexanoic, octanoic, and decanoic acids are just some other examples of relevant metabolites for the food and cosmetic industry. To demonstrate that these products can also be conveniently monitored, we switched the polarity to negative ion mode. SESI-HRMS spectra in negative ion mode are dominated by deprotonated fatty acids^56,57^. Therefore, isomeric interferences such as esters could be excluded. Figure 3d shows the time traces of a complete series of 11 fatty acids detected in WT after injecting ^13^C_1_-glucose. Acetic, octanoic, and decanoic acids were the major products. The complete list of all 43 compounds detected in this experiment is given in supplementary Table S5.

Thus, in total, 8 compounds were identified with a high degree of confidence by real-time MS/MS, although isomeric structures cannot be excluded. 11 fatty acids were also confirmed in negative ion mode with a high degree of confidence based on prior studies^56,57^. Additional hints of potential compounds were sought by querying publicly available databases. Of the 263 peaks associated to glucose metabolism in positive ion mode, 111 formulae matched at least one compound present in the yeast metabolome database (Table S1)^58^.

By injecting ^13^C_1_-glucose, we also aimed to confirm the validity of our approach to capture well-known biological processes. For example, while the contribution of the oxidative pentose phosphate pathway is rather small in baker’s yeast grown on glucose, this little contribution should be detectable^59^. This is indeed suggested in Fig. 3c, which shows the normalized isotopic distributions for farnesene for the three strains. The maximum isotope accumulation for all three strains was found at ^13^C_4_, but clearly, the relative abundance of C_5_ to C_9_ is greater in the culture of the *zwf1* knockout strain as no ^13^CO_2_ is released during breakdown of glucose to acetyl-CoA. The same trend was observed for acetic acid, ethanol, ethyl octanoate, ethyl decanoate, and octanoic acid. All these observations were reproduced in a replicate experiment (Figure S7).

As metabolic systems are highly plastic, the ability to monitor metabolite dynamics is crucial for understanding cellular processes. Here, our time-resolved data was pivotal for understanding the metabolic pathways involved in the metabolism of glucose that are favored by each yeast strain. Figure 3f shows the ^13^C/^12^C ratio for ethanol and acetic acid. Note that, in the case of ethanol, we monitored the dimer, which lead to a triplet-like isotopic distribution (i.e., *m/z* 93 for C_2_H_6_O-C_2_H_6_O; *m/z* 94 for C(^13^C)H_6_O-C_2_H_6_O and *m/z* 95 for C(^13^C)H_6_O-C(^13^C)H_6_O; inset in Fig. 3f). Both, the kinetic and isotopic ratios were different for the three strains. Mutant *zwf1* incorporated ^13^C into ethanol steeply to reach a plateau of ^13^C/^12^C ratio of ~1.2 after 5 h. The label incorporation in ethanol formed by the WT was similar but reached lower final fractional labeling. In contrast, no labeled ethanol was observed for *pfk1* in the first 4 h after the ^13^C tracer injection and did not reach a steady labeling within the 25 h of the experiment. This is explained by a slowed-down glucose metabolism and the delayed ethanol formation in this mutant. Interestingly, the ^13^C in acetic acid built up immediately upon glucose injection in all strains. Other compounds showed different kinetics (Figure S8). The time resolution capability allowed to exquisitely capture fine details of kinetic profiles, which is crucial to adjust industrial parameters to tune in real-time the desired volatile profile.

In conclusion, we deployed a sensitive and selective, yet, real-time mass spectrometric technique to investigate the production of volatile metabolites during yeast growth. The technique gently tracks biological processes at the metabolic level *in vivo* with a time resolution of less than one minute. Thus, we benchmarked the technique by observing well-known processes such as production of ethanol from glucose. However, despite being one of the most widely studied organisms, the rich volatile profiles (~300 metabolites combining positive and negative mode) of *S*. *cerevisiae* detected in these analyses, including non-reported analytes suggests that much work remains to be accomplished to fully map the metabolism of yeast. Such comprehensive metabolic coverage may also have potential to tune industrial processes where yeast fermentation is involved.

## Methods

### Secondary Electrospray Ionization-High Resolution Mass Spectrometry (SESI-HRMS)

SESI-HRMS experiments were carried out using a commercial ion source (SEADM S.L.)^60^ plugged onto a Thermo-Fisher LTQ Orbitrap mass spectrometer. The SESI solvent was 0.1% formic acid in water infused at ~100 nL/min through a 20 μm ID silica capillary. The electrospray voltage was set to 5.4 kV in positive mode (i.e., focusing electrode 2.59 kV and impact electrode 1.6 kV) and to 5 kV in negative mode. The sweep gas used for cleaning the electrospray region was set to 2 a.u. All other internal Orbitrap parameters were optimized during calibration. Orbitrap scan parameters: scan type: FTMS full MS [50–500 Da]; source fragmentation: off; resolution: 30.000; polarity: positive and negative; Typical mass accuracies were within 2 ppm by using common chemical noise encountered in SESI-HRMS background as lock masses (i.e. m/z 149.0233, 279.1591 and 445.1200)^61,62^. AGC target: 30.000; Maximum injection time: 150 ms; micro scans: 30, which led to acquire a profile mass spectrum every ~49 seconds to prevent the generation of intractable large files during several hours of volatiles monitoring. The system was properly calibrated in the respective polarities prior to the analysis. MS/MS fragmentation spectra for both, yeast and standards, were obtained under identical conditions: Same collision energies; 5 microscans; Isolation width: 1 Da; Activation Q: 0.25 and Activation time: 30 ms.

### Yeast cell cultures

The bioreactor consisted of an autoclaved 100 mL three-neck flask filled with 20 mL of medium, stirred at 800 rpm with a Teflon magnet and uniformly heated at 40 °C in a water bath. The metabolites produced during yeast growth were dragged downstream towards the SESI source by a continuous flow of compressed air at 0.5 L/min. The compressed air was filtered and humidified upstream the bioreactor. The bioreactor was connected to the ion source through a stainless-steel tube (OD 6 mm) heated at 130 °C to minimize metabolite adsorption onto the tube walls.

All the described experiments in this study were performed with Saccharomyces cerevisiae, prototrophic, YSBN.6 (wild-type) strain. Cells were grown in minimal defined medium: BD (DIFCO) yeast nitrogen base (#233520); and 2% glucose (as only carbon-source). For each experiment, pre-cultures were inoculated from SD plates and grown at 30 °C while shaking with 300 rpm for 8 to 10 hours in 1 mL pre-culture tubes. Then, the cells were inoculated (starting optical density; OD ~0.1) in 500 mL Erlenmeyer flasks with 50 mL of growth medium. Cultivation was performed at 30 °C with stirrer bar at 300 rpm until an OD of 1.2. The YSBN.6 strains *zwf1*Δ (Δzwf::Kan) and *pfk1*Δ (Δ*PFK1*::Kan), were grown in a similar minimal defined medium with the addition of 0.02% (v/v) of kanamycin and under the same time, temperature, and mixing conditions. To start the experiment, 1 mL of inoculum with an OD (between 0.8 to 1.0) was injected into the bioreactor for obtaining a starting OD of 0.1. When the ethanol signal decayed, 0.8 mL of 50% glucose was injected to prolong the exponential growth phase.

During the experiment, a time-lapse camera took a picture of the bioreactor every minute. Using ImageJ, an open platform for scientific image analysis^63^, yeast concentrations of relative values were obtained by averaging grayscale level in a region of interest of the image in a video. Absolute OD values were also obtained at the beginning and at the end of each experiment with a Thermo electron corporation GENESYS 10 UV *spectrophotometer*.

**Figure 4:**
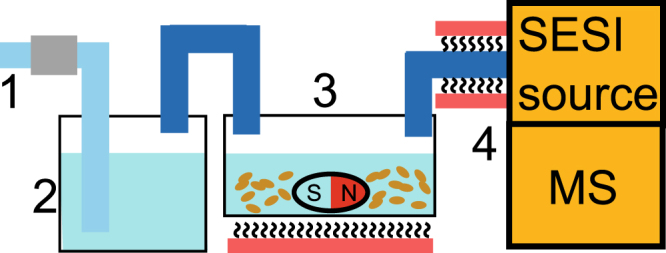
Experimental set-up: 0.5 L/min of compressed air flowed constantly through an active carbon filter (1); it was humidified in a gas washing bottle (2) before entering the bioreactor (3), a three-neck bottle with rubber stoppers filled with 50 mL of medium, heated to 30 °C and stirred at 800 rpm. The gas-phase metabolites were dragged to the SESI source (4) to be analyzed in the mass spectrometer.

Figure 4 shows a schematic of the experimental set-up (photograph in Figure S9).

### Data analysis

The raw mass spectra were transformed into mzXML format via MSConvert (Proteowizard)^64^ and imported to MATLAB (R2016b). Each sample file was interpolated linearly (10^6^ points in the range 50–500 Da). These interpolated profile mass spectra were then centroided (intensity threshold of 50 a.u.), resulting in ~4,400 mass spectral features (number of peaks dependent of course on the experiment performed). Subsequently, we computed the time traces for each of these peaks by adding the signal intensities ± 2, 5 mDa around the peaks. The resulting time traces (i.e., signal intensity for each *m/z* value as a function of time) were subjected to an agglomerative hierarchical cluster tree (Ward method; Euclidean distance; Figs 1c and 2d). To ease visualization, the time traces were smoothed (moving mean; span = 25). The 263 features finally considered (Table S1) were retained as they raised upon the ^12^C-glucose and ^13^C-glucose injection but did not experience any temporal change in a blank experiment were glucose was spiked in sterile fermentation medium. The signal to noise ratios were determined as the ratio of the variances of the signals of the experiment with yeast and the negative control where we spiked glucose into sterile medium (Figure S1).

The generation of molecular formulae from accurate mass was performed assuming protonated ions in the positive ion mode and deprotonated ions in negative mode. The following rules^65^ were taken into account: Masses up to 500 Da; considered elements were restricted to C, H, N and O; restricted maximum number of each of these elements, bounded H/C ratio and heteroatom/C ratio and limited number of double-bond equivalent.

Principal component analysis (PCA) was also used for dimensionality reduction and visualization of the mass spectra (Fig. 3a,b). The matrix subjected for PCA consisted of 30 rows (i.e., ten time points during the exponential growth for each of the three strains investigated) and 636 variables (i.e., peaks listed in Table S2). The data was mean-centered prior PCA. No further transformation of the matrix was applied.

## Electronic supplementary material

Supporting information
