# Supplementary material for: Comprehensive Real-Time Analysis of the Yeast Volatilome
Source: Sci Rep. 2017 Oct 27;7:14236. doi: 10.1038/s41598-017-14554-y (PMC5660155; doi:10.1038/s41598-017-14554-y)
Supplement: Supplementary file 1 — Supporting information [file 41598_2017_14554_MOESM1_ESM.pdf]

Supporting Information

Comprehensive Real-Time Analysis of the Yeast Volatilome

Alberto Tejero Rioseras[1,2,3], Diego Garcia Gomez[1,3], Birgitta E. Ebert[4], Lars M. Blank[4], Alfredo J. Ibáñez[1,5,*], Pablo M-L Sinues[1,6,*]

1) Department of Chemistry and Applied Biosciences, ETH Zurich, 8093 Zurich (Switzerland)
2) SEADM, S.L. (Spain)
3) Department of Analytical Chemistry, University of Cordoba (Spain)
4) Institute of Applied Microbiology – iAMB, Aachen Biology and Biotechnology – ABBt, RWTH Aachen University, Worringerweg 1, Aachen 52074 (Germany)
5) Instituto de Ciencias Ómicas y Biotecnología Aplicada - Pontificia Universidad Católica del Perú (ICOBA-PUCP), Lima (Peru)
6) University Children’s Hospital Basel, University of Basel (Switzerland)

*E-mail: aibanez@pucp.edu.pe
*E-mail: pablo.mlsinues@ukbb.ch

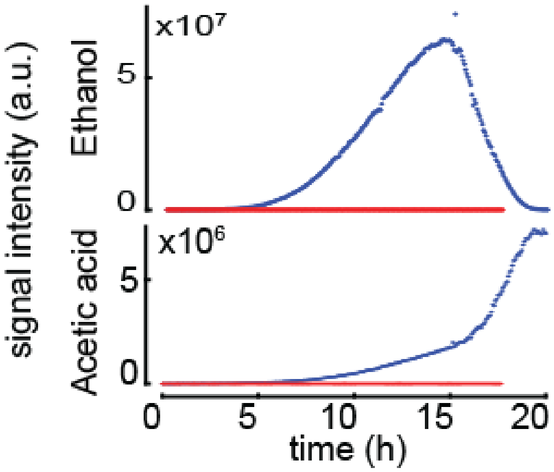

Figure S1. Signal intensity of ethanol and acetic acid after $^{13}C_6$-glucose injection in a solution containing yeast (same as Figure 1b; blue) and a negative control (red) under exactly the same conditions, but without yeast. As expected, ethanol and acetic acid production can be attributed to yeast growth.

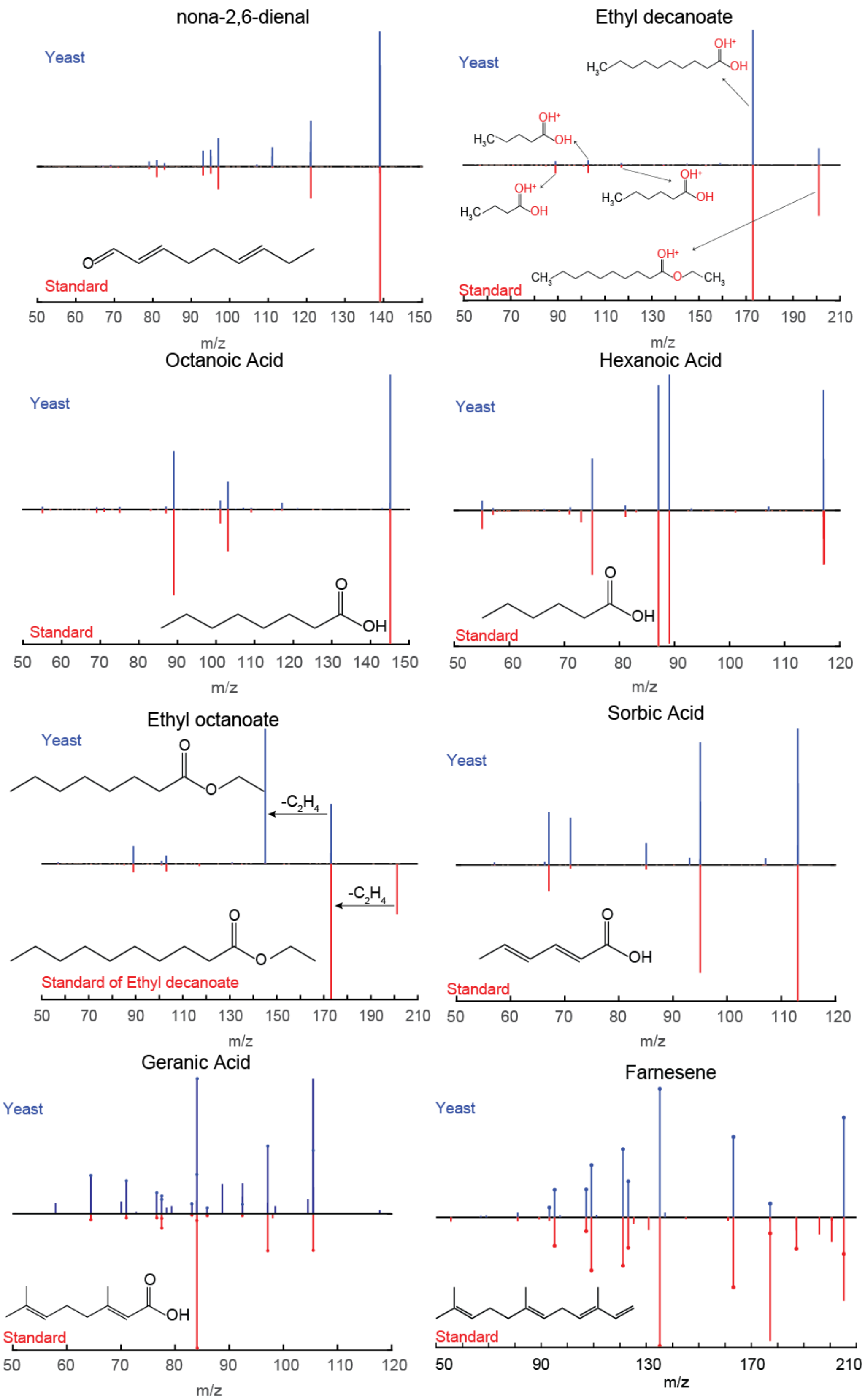

Figure S2. Head-to-tail MS/MS mass spectra of selected yeast metabolites (top) and standards (bottom).

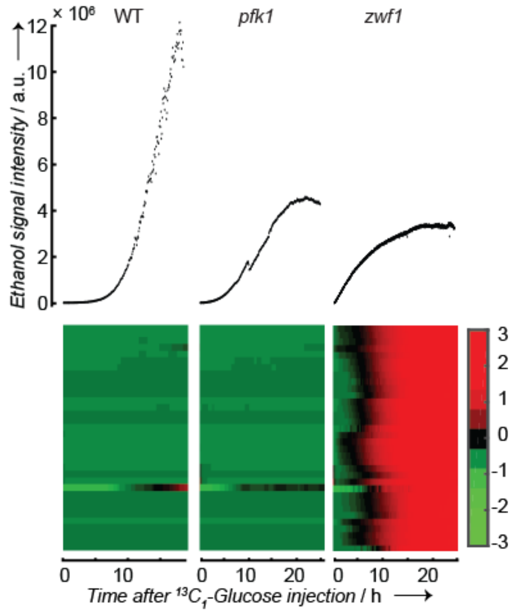

Figure S3. Replicated experiment injecting $^{13}C_1$-glucose to the cultivations of the three yeast strains WT, *pfk1, zwf1* provided a similar global picture as that shown in figure 2 of the main text, including ethanol (top traces) and a series of odd-numbered carbon compounds (i.e., $C_9H_{18}O$, $C_{11}H_{22}O$, $C_{13}H_{26}O$ and $C_{15}H_{30}O$) characteristic of *zwf1* (heatmap).

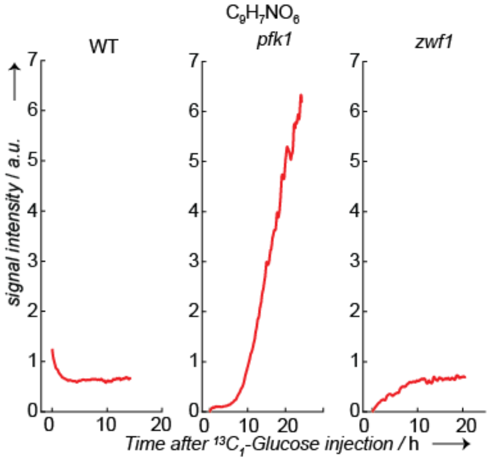

Figure S4. One selected compound detected in excess in mutant *pfk1*.

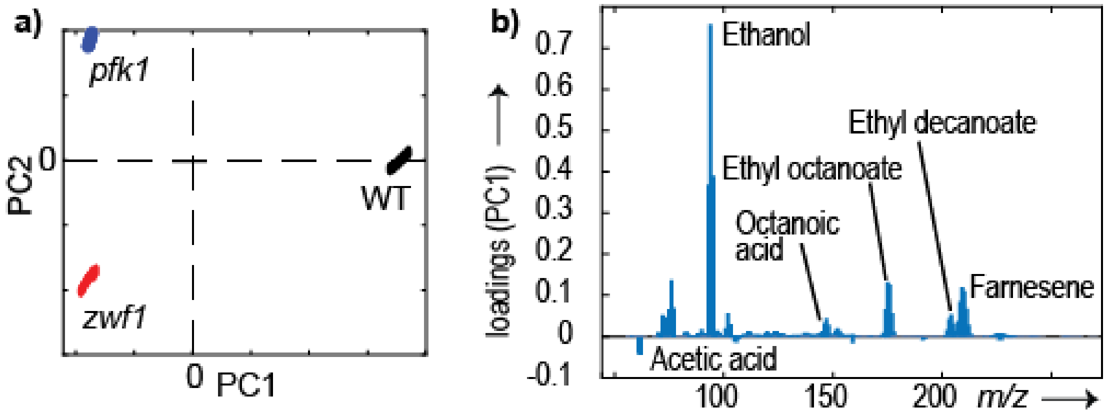

Figure S5. PCA including ethanol and acetic acid.

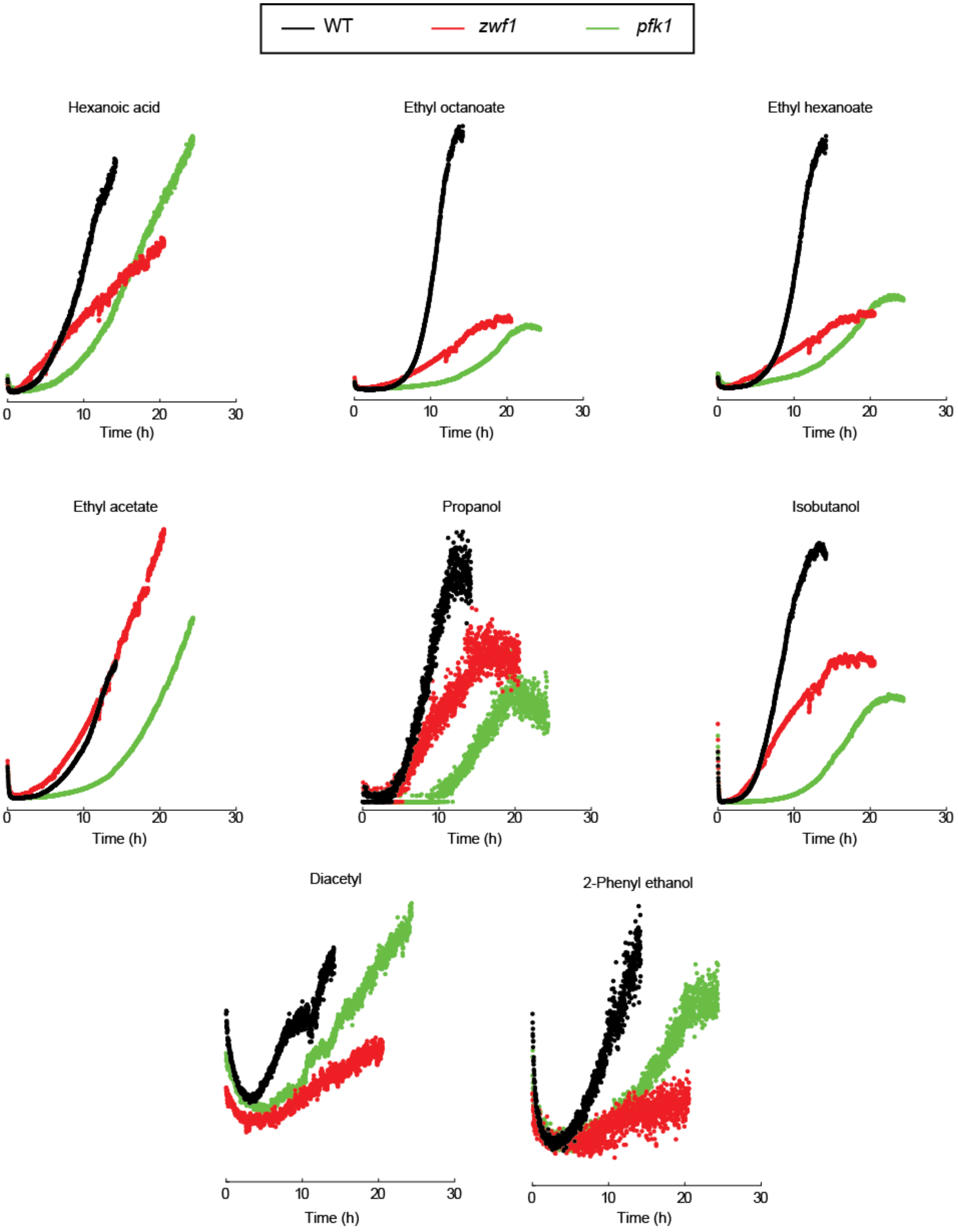

Figure S6. Aroma-relevant compounds were produced at different rates for the three strains investigated. Note that the bottom five compounds were not confirmed via MS/MS and thus assignment is only based on accurate mass and isotopic distribution.

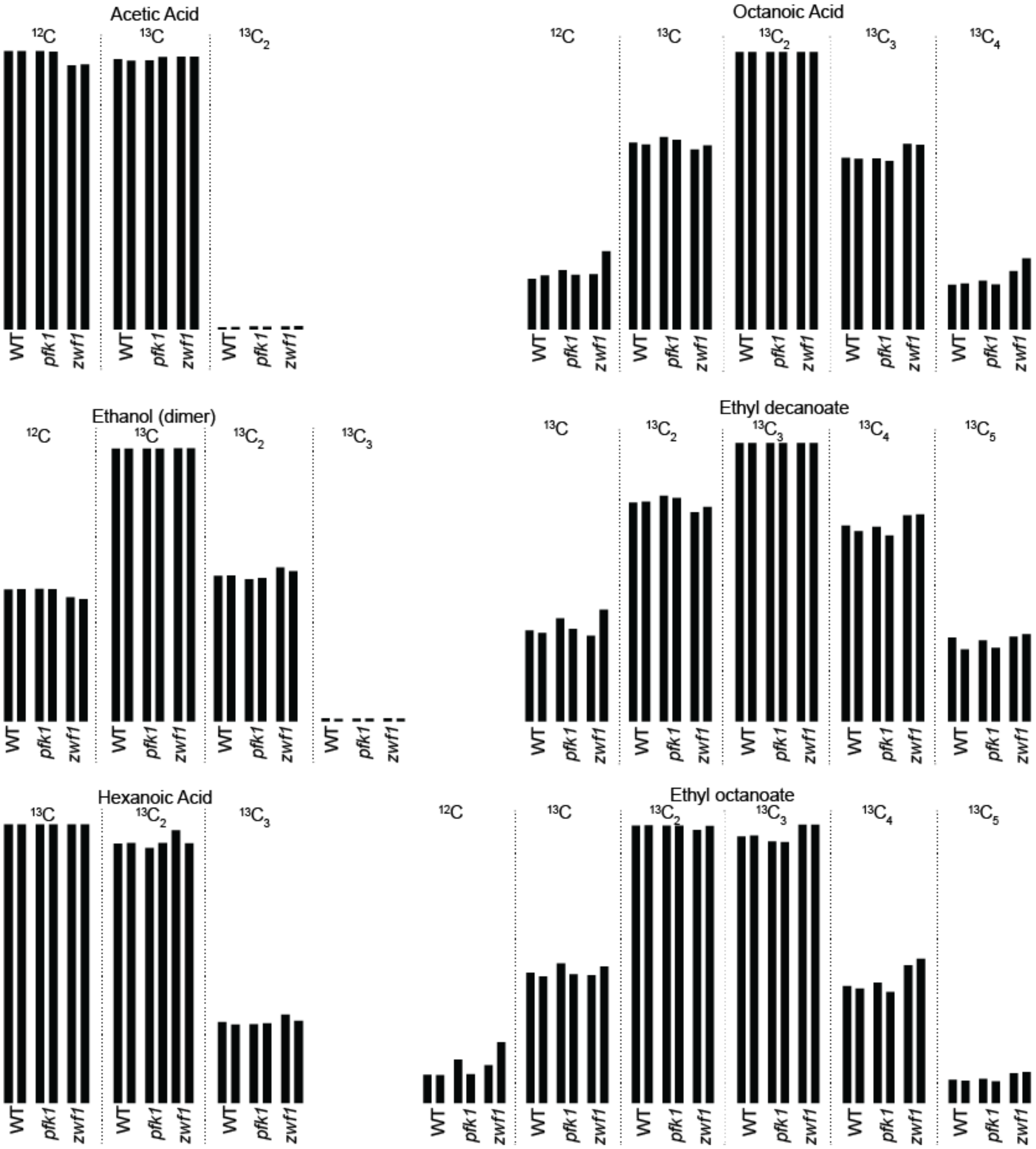

Figure S7. Replicate experiment confirmed enhanced incorporation of ^{13}C in metabolites of *zwf1* as compared to WT and *pfk1*.

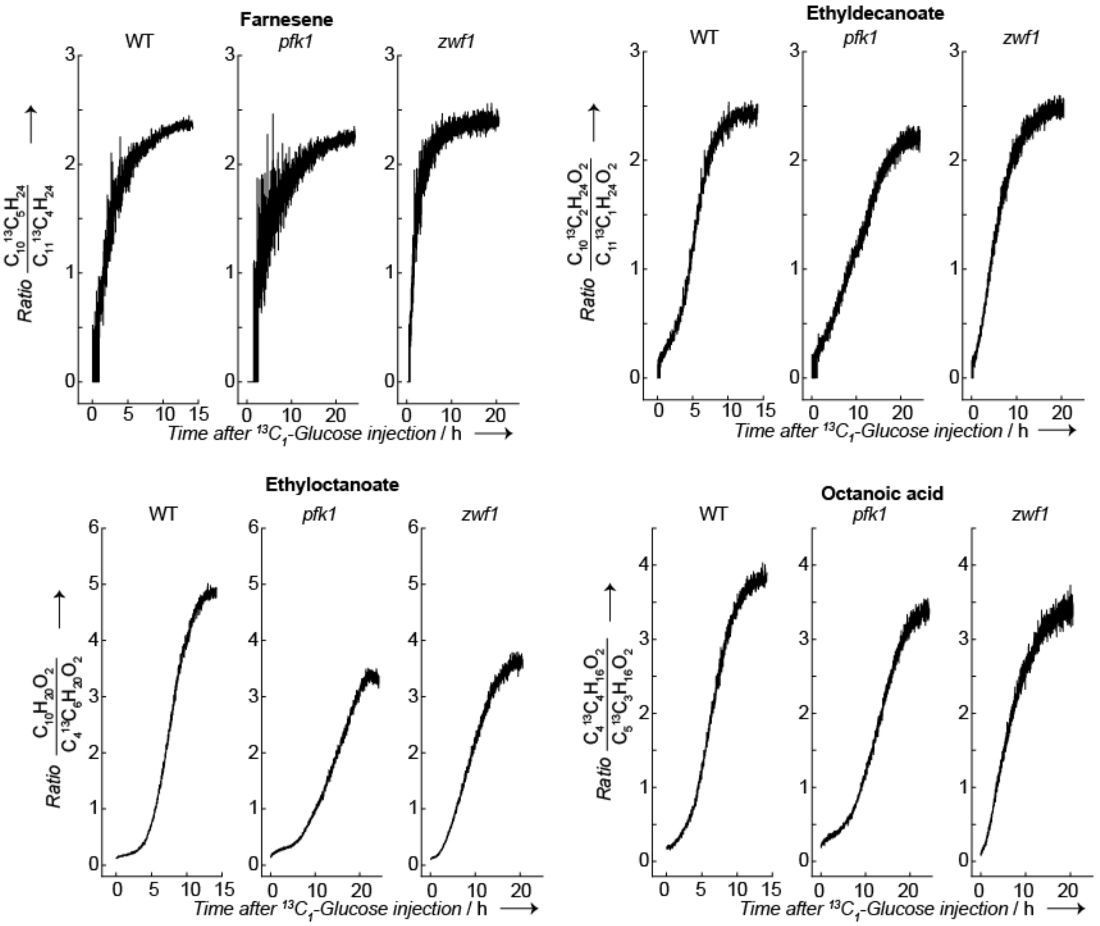

Figure S8. $^{13}C/^{12}C$ ratios for identified compounds show the kinetics of volatiles production upon $^{13}C_1$-glucose injection.

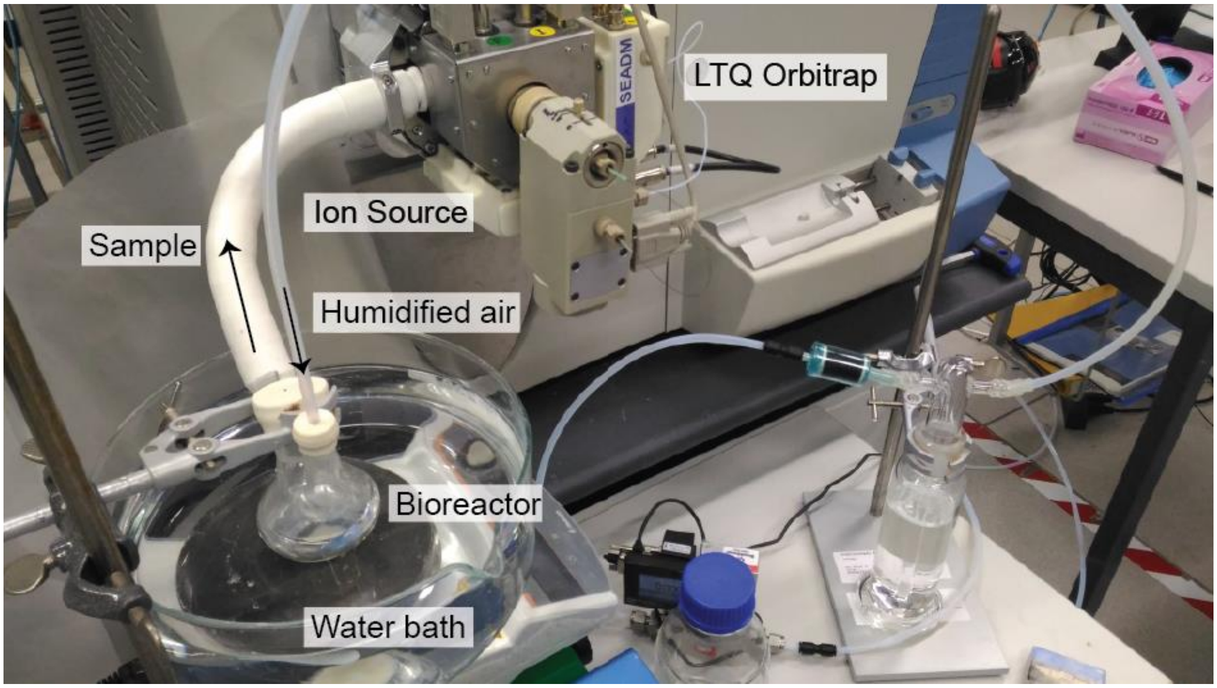

Figure S9. Experimental set-up used to monitor yeast volatiles during growth in glucose.

Table S1 263 signals detected upon injecting $^{13}C_6$-glucose and ^{12}C-glucose to WT. The order corresponds to the one shown in the heatmap from Figure 1b (top-to-bottom). Note how series of molecules with similar molecular formula tend to cluster together. The molecular *formulae* were generated assuming $[M+H]^+$ ion formation.

#	Molecular formula	Match with databases*		*m/z* measured with ^{12}C-glucose	error (ppm)	*m/z* measured with $^{13}C_6$-glucose	Mass error (ppm)	RDBE	S/N**
**1**	C2H6O2	YMDB01564	Ethylene glycol	63.0440	-0.2	65.0506	-1.8	0	4.0E+03
**2**	C10H10O2	YMDB01744	Methyl cinnamate	163.0754	0.1	173.1088	-0.4	6	5.1E+04
**3**	C8H6O	HMDB32929	Benzofuran	119.0486	-4.8	127.0754	-4.3	6	7.3E+02
**4**	C8H8N2	HMDB33171	1-Methylpyrrolo[1,2-a]pyrazine	133.0759	-0.8	141.1032	2.6	6	2.8E+02
**5**	C8H8O4	YMDB01802	Vanillic acid	169.0495	-0.4	177.0763	-0.3	5	6.1E+00
**6**	C11H10O3	HMDB40938	Coixinden A	191.0705	1.1	202.1074	1.5	7	3.4E+01
**7**	C8H3NO2	HMDB31004	Diatretin 2	146.0236	-0.5	154.0500	-3.2	8	2.2E+01
**8**	C11H9NO2	HMDB00734	Indoleacrylic acid	188.0707	0.3	199.1076	0.8	8	2.1E+01
**9**	C11H9NO			172.0757	0.1	183.1126	0.6	8	8.2E+00
**10**	C11H11NO	HMDB32887	4-Amino-2-methyl-1-naphthol	174.0908	-3.1	185.1286	2.5	7	5.0E+01
**11**	C12H9NO	JP007862	PHENYL 2-PYRIDYL KETONE	184.0754	-1.6	196.1163	2.1	9	4.0E+01
**12**	C11H5NO			168.0450	3.9	179.0820	4.2	10	2.6E+02
**13**	C8H8N2O2	HMDB42006	Ricinine	165.0658	-0.1	173.0922	-2.5	6	7.4E+01
**14**	C19H24			253.1951	0.3	272.2580	-2.6	8	6.7E+00
**15**	C8H8O3	YMDB01803	Vanillin	153.0545	-0.8	161.0809	-3.4	5	1.4E+02
**16**	C8H7NO5	HMDB62403	4-hydroxy-3-nitrophenylacetate	198.0396	-0.6	206.0655	-4.9	6	1.0E+02
**17**	C9H6O5			195.0286	-1.2	204.0585	-2.4	7	1.7E+03
**18**	C14H10O3	HMDB33925	Oroselone	227.0700	-1.2	241.1180	3.2	10	4.0E+01
**19**	C14H12O3	YMDB01780	Resveratrol	229.0860	0.4	243.1335	2.8	9	2.8E+00
**20**	C11H14	HMDB61808	(3-Methyl-2-butenyl)-benzene	147.1167	-0.8	158.1541	2.6	5	1.2E+04
**21**	C7H19N5O4			238.1517	3.2	245.1746	0.7	1	4.9E+01
**22**	C11H10O2	HMDB31845	3-Propylidene-1(3H)-isobenzofuranone	175.0755	0.9	186.1125	1.3	7	2.5E+01
**23**	C11H9NO3	YMDB00169	3-(indol-3-yl)pyruvic acid	204.0656	0.5	215.1026	0.9	8	2.4E+00
**24**	C11H8O5	HMDB30789	Dracunculin	221.0444	-0.3	232.0818	2.1	8	6.6E+00
**25**	C8H11NO4			186.0762	0.4	194.1021	-4.1	4	1.5E+00
**26**	C8H3NO			130.0282	-4.4	138.0550	-3.9	8	2.5E+01

**27**	C7H14O	YMDB01337	2-Heptanone	115.1116	-1.7	122.1353	0.9	1	Inf
**28**	C6H12	HMDB29597	Cyclohexane	85.1013	2.0	91.1211	-1.6	1	1.3E+06
**29**	C19H28O2	HMDB36205	alpha-Amylcinnamyl isovalerate	289.2162	-0.1	308.2795	-1.2	6	2.1E+04
**30**	C10H16O3	HMDB36998	(S)-Oleuropeic acid	185.1174	1.2	195.1509	0.7	3	2.7E+02
**31**	C10H18O3	YMDB16206	3-Oxodecanoic acid	187.1330	0.7	197.1664	0.2	2	3.5E+04
**32**	C10H18O	YMDB01760	Nerol	155.1431	0.2	165.1765	-0.3	2	Inf
**33**	C5H8	C16521	Isoprene	69.0701	3.3	74.0868	2.5	2	9.0E+07
**34**	C7H6			91.0544	1.7	98.0777	0.0	5	1.0E+07
**35**	C9H3NO3			174.0187	0.7	183.0481	-3.2	9	7.7E+01
**36**	C9H10O3	YMDB01687	Ethyl vanillin	167.0702	-0.4	176.1001	-1.9	5	7.5E+01
**37**	C5H8O3	YMDB00365	Alpha-Ketoisovaleric acid	117.0545	-0.8	122.0712	-1.1	2	5.3E+02
**38**	C11H22O	YMDB01592	2-Undecanone	171.1743	-0.1	182.2113	0.4	1	5.8E+05
**39**	C16H32O2	YMDB00069	Palmitic acid	257.2478	1.0	273.3014	1.1	1	2.5E+04
**40**	C7H16O2	HMDB37636	xi-1-Butoxy-1-methoxyethane	133.1225	1.6	140.1458	0.4	0	5.0E+07
**41**	C4H10O	YMDB00573	isobutanol	75.0805	0.4	79.0941	3.4	0	3.3E+14
**42**	C4H12O2	YMDB00883	Ethanol (dimer)	93.0910	-0.2	97.1046	2.2	-1	4.2E+10
**43**	C3H8O	YMDB01718	Isopropanol	61.0648	-0.2	64.0749	0.8	0	6.2E+06
**44**	C5H14O2			107.1067	0.2	112.1234	-0.2	-1	3.5E+06
**45**	C12H24O2	YMDB01391	Ethyl decanoate	201.1854	2.6	213.2250	-0.5	1	6.3E+08
**46**	C9H24N4O			205.2027	1.8	214.2317	-3.7	0	3.5E+11
**47**	C5H10	JP005997	Cyclopentane	71.0857	1.8	76.1024	1.1	1	Inf
**48**	C7H18O2			135.1381	0.8	142.1614	-0.2	-1	2.3E+07
**49**	C14H28O2	YMDB01332	Ethyl dodecanoate	229.2164	0.7	243.2634	1.3	1	2.8E+07
**50**	C6H14O2	YMDB01709	Hexanediol	119.1068	1.4	125.1266	-1.2	0	Inf
**51**	C4H6N2O	HMDB60768	4-Hydroxymethylpyrazole	99.0557	3.8	103.0689	1.6	3	3.8E+04
**52**	C4H10N2O			103.0868	1.8	107.1004	3.9	1	Inf
**53**	C4H9N	HMDB31641	Pyrrolidine	72.0807	-1.6	76.0943	1.6	1	Inf
**54**	C17H30O	HMDB31336	(Z)-9-Cycloheptadecen-1-one	251.2374	1.8	268.2946	2.5	3	Inf
**55**	C18H28O2	HMDB06547	Stearidonic acid	277.2165	1.0	295.2767	0.7	5	Inf
**56**	C10H23NO2			190.1803	0.8	200.2137	0.3	0	3.8E+04
**57**	C11H22O2	YMDB01351	Isopentyl hexanoate	187.1693	0.2	198.2062	0.6	1	Inf

**58**	C10H20O2	YMDB01389	Ethyl octanoate	173.1540	2.5	183.1875	1.9	1	Inf
**59**	C6H16O2	YMDB01718	Isopropanol (dimer)	121.1224	0.6	127.1426	1.6	-1	3.1E+03
**60**	C13H26O2	YMDB01364	Propyl decanoate	215.2007	0.6	228.2438	-1.5	1	9.5E+02
**61**	C12H27NO2			218.2117	1.1	230.2517	0.2	0	Inf
**62**	C18H31NO2			294.2427	-0.1	312.3021	-3.1	4	4.3E+01
**63**	C5H15N5O2			178.1304	3.3	183.1458	-4.4	1	9.0E+02
**64**	C20H38O4	JP001741	Octanedioic acid, dihexyl ester	343.2845	0.5	363.3522	2.5	2	Inf
**65**	C13H22N2O5			287.1612	3.7	300.2034	-1.0	4	1.1E+02
**66**	C18H34O4	HMDB00782	Octadecanedioic acid	315.2531	0.2	333.3138	1.4	2	1.1E+13
**67**	C12H20			165.1640	1.1	177.2040	-0.1	3	5.4E+04
**68**	C5H4			65.0385	-0.5	70.0553	-1.1	4	Inf
**69**	C8H16O2	YMDB00676	Octanoic acid	145.1222	-0.7	153.1490	-0.5	1	4.6E+09
**70**	C18H34O2	YMDB01368	Ethyl 9-hexadecenoate	283.2636	1.5	301.3238	1.3	2	Inf
**71**	C10H5NO5			220.0243	1.1	230.0568	-3.2	9	3.2E+04
**72**	C10H5NO2			172.0394	0.7	182.0719	-4.8	9	5.4E+02
**73**	C13H20	HMDB61825	1-Phenylheptane	177.1636	-0.7	190.2076	1.6	4	9.0E+05
**74**	C15H22	YMDB16091	alpha-Curcumene	203.1795	0.3	218.2296	-0.3	5	Inf
**75**	C15H24O	YMDB15971	Butylated hydroxytoluene	221.1900	0.0	236.2401	-0.5	4	Inf
**76**	C7H12			97.1010	-1.4	104.1248	1.6	2	Inf
**77**	C8H14O	YMDB01444	1-octen-3-one	127.1117	-0.4	135.1385	-0.2	2	Inf
**78**	C9H16	HMDB37777	2-Isopropyl-1,4-hexadiene	125.1324	-0.4	134.1628	1.1	2	Inf
**79**	C8H14	YMDB15921	1,3-Octadiene	111.1167	-0.9	119.1436	-0.6	2	Inf
**80**	C6H10	HMDB31544	3-Methylcyclopentene	83.0858	3.2	89.1056	-0.6	2	6.3E+08
**81**	C6H6	HMDB01505	Benzene	79.0542	0.1	85.0745	1.7	4	4.5E+05
**82**	C7H8	HMDB34168	Toluene	93.0699	0.6	100.0932	-0.9	4	2.1E+10
**83**	C12H18	HMDB13806	1,3-Diisopropylbenzene	163.1484	1.7	175.1884	0.5	4	7.9E+07
**84**	C15H29NO			240.2323	0.3	255.2828	1.5	2	Inf
**85**	C15H27N1			222.2222	2.5	237.2719	0.0	3	Inf
**86**	C7H10	HMDB31532	1-Methyl-1,3-cyclohexadiene	95.0855	-0.5	102.1092	2.6	3	6.4E+10
**87**	C8H12	HMDB61897	(Z)-1,3-Octadiene	109.1012	0.0	117.1280	0.2	3	Inf
**88**	C8H10	YMDB16013	Ethylbenzene	107.0856	0.9	115.1124	1.1	4	6.9E+04

**89**	C15H24	YMDB16101	beta-Farnesene	205.1950	-0.2	220.2461	3.3	4	Inf
**90**	C11H16	HMDB61809	1-Methyl-4-(1-methylpropyl)-benzene	149.1327	1.5	160.1696	2.0	4	2.6E+09
**91**	C9H12	YMDB15979	Cumene	121.1013	1.2	130.1312	-0.9	4	3.7E+06
**92**	C15H26O	YMDB16053	Nerolidol	223.2060	1.6	238.2561	1.0	3	Inf
**93**	C9H14	HMDB38140	Santene	123.1169	0.4	132.1472	1.8	3	Inf
**94**	C10H14	YMDB16062	P-Cymene	135.1170	1.4	145.1504	0.7	4	Inf
**95**	C6H8			81.0698	-1.1	87.0900	0.5	3	Inf
**96**	C4H10O2	YMDB01407	2,3-butanediol	91.0754	0.8	95.0886	-1.4	0	7.2E+03
**97**	C2H5NO	YMDB01563	Acetamide	60.0447	4.9	62.0513	3.0	1	2.5E+08
**98**	C5H6	HMDB61878	Cyclopentadiene	67.0545	4.8	72.0713	3.9	3	1.7E+07
**99**	C10H5NO4			204.0289	-1.2	214.0623	-1.5	9	1.9E+05
**100**	C10H5NO3			188.0344	0.8	198.0669	-4.2	9	1.9E+07
**101**	C10H7NO2	HMDB00842	Quinaldic acid	174.0550	0.1	184.0879	-2.8	8	6.4E+03
**102**	C10H7NO	HMDB62189	1-nitrosonaphthalene	158.0600	-0.2	168.0939	2.0	8	5.9E+02
**103**	C10H16	YMDB01753	Myrcene	137.1326	0.7	147.1660	0.0	3	Inf
**104**	C11H10O4	HMDB29775	Vinyl caffeate	207.0654	1.2	218.1019	-0.5	7	2.5E+00
**105**	C9H10O	YMDB01619	4-Methylacetophenone	135.0803	-1.2	144.1102	-2.9	5	1.6E+02
**106**	C8H10O3	YMDB16081	Syringol	155.0701	-1.4	163.0964	-3.9	4	1.3E+00
**107**	C8H10O2	YMDB01798	Tyrosol	139.0751	-1.9	147.1015	-4.7	4	7.1E+01
**108**	C5H6O2	YMDB15931	2-Furanmethanol	99.0440	-0.4	104.0607	-0.8	3	2.7E+04
**109**	C5H8O4	YMDB00394	2-Acetolactate	133.0495	-0.3	138.0658	-3.9	2	5.3E+01
**110**	C4H10O3	HMDB61944	Trihydroxybutane	107.0704	1.1	111.0836	-0.8	0	9.8E+02
**111**	C7H15NO2	HMDB06831	3-Dehydroxycarnitine	146.1177	0.7	153.1405	-3.2	1	7.7E+04
**112**	C7H14	HMDB61895	2,4-Dimethyl-2-pentene	99.1166	-2.4	106.1403	0.6	1	Inf
**113**	C9H18O3	HMDB31513	(±)-3-Hydroxynonanoic acid	175.1329	-0.1	184.1632	1.0	1	4.3E+06
**114**	C13H22			179.1796	1.2	192.2227	-1.3	3	1.2E+04
**115**	C15H26O2	HMDB40281	Geranyl valerate	239.2005	-0.2	254.2506	-0.7	3	6.2E+04
**116**	C9H16O	YMDB01794	trans-2-Nonenal	141.1274	0.0	150.1573	-1.7	2	1.9E+07
**117**	C15H28O	HMDB32218	(+/-)-Dihydrofarnesol	225.2216	1.2	240.2712	-1.3	2	Inf
**118**	C10H18	HMDB61795	(S)-3,7-Dimethyl-1,6-octadiene	139.1481	0.0	149.1815	-0.6	2	Inf
**119**	C6H12O	YMDB01421	trans-3-hexen-1-ol	101.0959	-2.3	107.1161	-1.0	1	Inf

**120**	C11H20O	YMDB01588	2-Methylisoborneol	169.1588	0.5	180.1957	1.0	2	Inf
**121**	C12H12	HMDB59764	2,6-Dimethyl-naphtalene	157.1008	-2.1	169.1413	-0.4	7	1.5E+02
**122**	C14H24	HMDB61832	1-Acetyl-2-methylcyclopentene	193.1953	1.4	207.2419	-0.1	3	Inf
**123**	C9H10	YMDB16095	alpha-Methylstyrene	119.0858	2.0	128.1152	-3.6	5	1.2E+04
**124**	C6H4	YMDB01430	2-methoxyphenol	77.0387	1.4	83.0589	2.9	5	2.6E+02
**125**	C15H26			207.2106	-0.7	222.2612	0.8	3	Inf
**126**	C15H30O2	YMDB16051	Methyl tetradecanoate	243.2321	0.9	258.2817	-1.4	1	9.1E+08
**127**	C11H18	HMDB61811	4-Methyl-2-methylene-1-(1methylethylidene)-cyclohexane	151.1483	0.9	162.1848	-1.4	3	1.7E+08
**128**	C13H24	HMDB41081	(Z)-1,5-Tridecadiene	181.1952	0.7	194.2387	0.6	2	Inf
**129**	C9H18O2	YMDB01347	Propyl hexanoate	159.1379	-0.4	168.1678	-1.8	1	Inf
**130**	C10H16O	YMDB01455	trans,trans-2,4-decadienal	153.1275	0.9	163.1609	0.3	3	Inf
**131**	C9H16O2	YMDB01605	3-Methyl-4-octanolide	157.1223	0.3	166.1522	-1.3	2	1.8E+05
**132**	C15H20	HMDB59858	beta-Calacorene	201.1639	0.7	216.2145	2.2	6	Inf
**133**	C4H6	HMDB41792	1,3-Butadiene	55.0544	3.3	59.0676	-0.6	2	8.6E+06
**134**	C12H16	HMDB61814	1-(1-methylethenyl)-3-(1-methylethyl)-benzene	161.1324	-0.5	173.1729	1.1	5	1.1E+04
**135**	C20H34O	HMDB39827	Serratol	291.2685	0.8	311.3357	1.6	4	2.2E+04
**136**	C16H26	HMDB34498	(3E,7E)-4,8,12-Trimethyl-1,3,7,11-tridecatetraene	219.2107	0.0	235.2648	2.1	4	Inf
**137**	C20H32	HMDB36845	Cembrene	273.2580	1.0	293.3248	0.4	5	Inf
**138**	C10H16O2	HMDB36103	Geranic acid	169.1220	-1.6	179.1555	-2.0	3	Inf
**139**	C8H12O3	HMDB32232	2,5-Dimethyl-4-ethoxy-3(2H)-furanone	157.0861	0.9	165.1129	1.0	3	3.7E+06
**140**	C10H14O	YMDB01648	Carvone	151.1115	-1.4	161.1454	0.9	4	Inf
**141**	C7H14O2	YMDB01342	Ethyl pentanoate	131.1065	-1.1	138.1298	-2.1	1	1.6E+06
**142**	C6H8O3	YMDB01694	Furaneol	129.0547	0.4	135.0749	1.3	3	3.1E+04
**143**	C8H9NO3	HMDB01545	Pyridoxal	168.0657	0.8	176.0916	-4.2	5	8.6E+00
**144**	C8H11NO3	HMDB04817	5-Hydroxydopamine	170.0812	0.2	178.1076	-2.2	4	1.1E+01
**145**	C16H28O	HMDB36831	Ambronide	237.2217	1.7	253.2753	1.8	3	Inf
**146**	C16H30O2	YMDB00131	Oleic acid	255.2322	1.4	271.2859	1.5	2	Inf
**147**	C11H14O3	YMDB01809	Zingerone	195.1016	0.1	206.1385	0.5	5	3.1E+01
**148**	C7H10O2	YMDB16038	Lavander lactone	127.0754	0.4	134.0987	-0.7	3	3.6E+04
**149**	C11H12O3	HMDB35275	Isomyristicin	193.0860	0.6	204.1230	1.0	6	7.7E+00

**150**	C3H8O3	YMDB00283	Glycerol	93.0547	0.8	96.0648	1.5	0	1.4E+03
**151**	C14H4N4O			245.0447	-4.6	259.0922	-2.0	15	1.6E+01
**152**	C18H36O2	YMDB01349	Ethyl hexadecanoate	285.2791	1.2	303.3394	0.9	1	Inf
**153**	C10H18O2	YMDB01611	3,7-Dimethyl-1,5-octadien-3,7-dio	171.1380	0.5	181.1715	0.0	2	8.3E+05
**154**	C7H12O2	YMDB01746	Methyl succinate	129.0910	-0.4	136.1138	-4.8	2	5.6E+03
**155**	C10H3NO4			202.0142	3.7	212.0463	-3.2	10	5.2E+05
**156**	C9H18O	YMDB01383	2-nonanone	143.1429	-0.7	152.1733	0.6	1	Inf
**157**	C7H12O	HMDB31540	4-Methylcyclohexanone	113.0960	-0.8	120.1193	-2.0	2	Inf
**158**	C10H12	YMDB15981	Dehydro-p-cymene	133.1010	-1.3	143.1344	-1.7	5	Inf
**159**	C9H14O	YMDB01795	trans-2,4-Nonaidenal / nona-2,6-dienal	139.1118	0.7	148.1417	-1.1	3	Inf
**160**	C6H8O	YMDB01437	2-ethylfuran	97.0648	-0.4	103.0850	0.9	3	1.5E+04
**161**	C12H22O2	YMDB01366	Ethyl 9-decenoate	199.1694	0.9	211.2090	-2.2	2	8.1E+05
**162**	C6H10O3	YMDB16200	3-Oxohexanoic acid	131.0702	-0.4	137.0905	0.6	2	1.6E+07
**163**	C6H8O2	YMDB01784	Sorbic acid	113.0597	0.0	119.0799	1.1	3	1.1E+05
**164**	C8H13NO	HMDB37868	2,4-Dimethyl-5-propyloxazole	140.1068	-1.1	148.1337	-0.9	3	2.2E+05
**165**	C14H24O	JP008396	Ethyl geranyl acetone	209.1903	1.5	223.2374	2.1	3	9.8E+05
**166**	C14H26O2	YMDB16214	trans-Tetra-dec-2-enoic acid	227.2008	1.1	241.2474	-0.1	2	2.0E+06
**167**	C5H13NO2			120.1018	-0.6	125.1185	-0.9	0	Inf
**168**	C10H12O2	YMDB01472	Phenylethyl acetate	165.0909	-0.5	175.1243	-0.9	5	Inf
**169**	C6H10O	YMDB01707	Hexa-2,4-dienol	99.0803	-1.4	105.1005	-0.1	2	Inf
**170**	C15H22O	HMDB36071	Dendrolasin	219.1744	0.5	234.2246	-0.1	5	3.3E+07
**171**	C15H20O	HMDB38147	Isogermafurene	217.1589	0.9	232.2090	0.3	6	2.7E+04
**172**	C11H16O	HMDB31866	2-Methyl-4-phenyl-2-butanol	165.1272	-1.1	176.1642	-0.5	4	4.4E+04
**173**	C13H18	HMDB61824	1-Methyl-4-(1-methyl-2-propenyl)-benzene	175.1481	-0.2	188.1916	-0.3	5	Inf
**174**	C11H12			145.1012	-0.2	156.1381	0.4	6	4.2E+03
**175**	C11H16O2	YMDB15988	Dihydroactinidiolide	181.1222	-0.7	192.1591	-0.2	4	2.1E+03
**176**	C10H14O2	YMDB15989	Dihydroeugenol	167.1065	-1.0	177.1399	-1.4	4	Inf
**177**	C9H14O3	HMDB30471	1,4-Ipomeadiol	171.1018	1.1	180.1316	-0.4	3	Inf
**178**	C16H24	HMDB36578	Cyperotundone	217.1952	0.4	233.2488	0.6	5	Inf
**179**	C6H15NO2			134.1175	-0.2	140.1378	0.7	0	Inf
**180**	C4H11NO2	YMDB01327	2-Amino-2-methyl-1,3-propanediol	106.0861	-1.1	110.0998	1.1	0	5.0E+05

**181**	C5H12O2	YMDB01423	3-ethoxy-1-propanol	105.0911	1.2	110.1078	0.7	0	1.1E+07
**182**	C6H12O3	YMDB01419	Ethyl 4-hydroxybutanoate	133.0858	-1.1	139.1060	-0.1	1	1.1E+07
**183**	C5H13NO3			136.0968	-0.2	141.1135	-0.5	0	4.1E+04
**184**	C5H10O3	YMDB01429	Ethyl 2-hydroxy propanoate	119.0705	2.2	124.0868	-1.8	1	3.8E+06
**185**	C5H8O2	YMDB01434	2,3-pentanedione	101.0600	3.1	106.0767	2.6	2	2.7E+09
**186**	C4H8O2	YMDB00410	(R)-Acetoin	89.0599	2.0	93.0731	-0.4	1	2.2E+07
**187**	C5H6O3	HMDB31859	Norfuraneol	115.0390	0.0	120.0557	-0.3	3	5.2E+05
**188**	C3H8O2	YMDB01405	propylene glycol	77.0597	0.4	80.0699	1.2	0	Inf
**189**	C3H6O	YMDB00911	Propanal	59.0492	1.4	62.0593	2.4	1	2.7E+06
**190**	C2H5NO2	YMDB00016	Glycine	76.0396	4.5	78.0462	3.0	1	4.5E+03
**191**	C15H24O2	HMDB02352	Capsidiol	237.1850	0.2	252.2355	1.5	4	7.1E+13
**192**	C14H22	HMDB59739	1-Ethyl-3,5-diisopropyl-benzene	191.1793	-0.5	205.2259	-1.9	4	Inf
**193**	C10H10O	YMDB01440	2-phenylbut-2-enal	147.0804	-0.2	157.1138	-0.7	6	2.3E+03
**194**	C16H26O	HMDB40179	2,6-Di-tert-butyl-4-ethylphenol	235.2057	0.2	251.2598	2.2	4	Inf
**195**	C16H28O2	HMDB00477	7Z,10Z-Hexadecadienoic acid	253.2162	0.0	269.2699	0.2	3	Inf
**196**	C5H10O	YMDB00485	2-Methylbutanal	87.0806	2.0	92.0973	1.4	1	Inf
**197**	C6H6O	YMDB16074	Phenol	95.0492	0.6	101.0694	1.8	4	8.9E+03
**198**	C10H12O	YMDB01637	Anethole	149.0960	-0.8	159.1294	-1.3	5	3.7E+04
**199**	C7H10O	HMDB40278	2-Isopropylfuran	111.0804	0.0	118.1037	-1.2	3	Inf
**200**	C8H14O2	YMDB01365	Ethyl 2-hexenoate	143.1067	0.0	151.1335	0.1	2	3.2E+06
**201**	C8H14O3	YMDB16204	3-Oxooctanoic acid	159.1016	0.3	167.1284	0.4	2	4.8E+05
**202**	C8H12O	YMDB15959	6-Methyl-3,5-heptadiene-2-one	125.0961	0.4	133.1230	0.5	3	1.2E+04
**203**	C7H8O	YMDB01730	m-Cresol	109.0649	0.9	116.0882	-0.4	4	Inf
**204**	C9H14O2	HMDB40285	Anapear	155.1068	0.9	164.1367	-0.7	3	2.4E+04
**205**	C11H14O	HMDB41493	4-Isopropylphenylacetaldehyde	163.1117	-0.5	174.1486	0.1	5	9.1E+02
**206**	C9H12O2	YMDB01615	4-Ethyl-2-methoxyphenol	153.0908	-1.4	162.1207	-2.9	4	7.4E+03
**207**	C12H14			159.1168	0.1	171.1569	-1.0	6	2.2E+04
**208**	C15H22O2	HMDB36427	Procurcumenol	235.1694	0.6	250.2195	0.1	5	1.2E+06
**209**	C9H12O	YMDB15966	Benzenepropanol	137.0958	-1.9	146.1262	-0.5	4	4.9E+03
**210**	C10H10			131.0855	-0.5	141.1193	2.1	6	Inf
**211**	C9H12O3	YMDB01712	Homovanillyl alcohol	169.0858	-1.0	178.1161	0.1	4	2.7E+03

**212**	C15H20O2	HMDB36688	Costunolide	233.1538	1.0	248.2044	2.3	6	6.7E+03
**213**	C10H12O3	HMDB11751	3-Methoxybenzenepropanoic acid	181.0863	2.3	191.1193	-0.6	5	1.4E+01
**214**	C10H14O3	YMDB16019	Isoamyl 2-furoate	183.1014	-0.7	193.1349	-1.1	4	4.5E+01
**215**	C20H38O2	HMDB35159	Paullinic acid	311.2950	1.7	331.3618	1.1	2	Inf
**216**	C15H31NO			242.2483	1.7	257.2984	1.1	1	1.7E+08
**217**	C16H33NO2			272.2589	1.9	288.3126	1.9	1	Inf
**218**	C18H32O2	YMDB00884	Linoleic acid	281.2480	1.9	299.3069	-2.9	3	3.3E+06
**219**	C8H16O	YMDB01352	1-Octen-3-ol	129.1272	-1.1	137.1545	2.4	1	4.4E+03
**220**	C9H10O2	YMDB01624	4-Vinylguaiacol	151.0752	-0.8	160.1051	-2.3	5	5.6E+01
**221**	C3H7NO2	YMDB00154	L-Alanine	90.0549	-0.8	93.0646	-4.9	1	1.6E+02
**222**	C4H4O3	HMDB32523	Succinic anhydride	101.0233	-0.4	105.0365	-2.4	3	3.9E+02
**223**	C10H20O3	YMDB16207	(R)-3-Hydroxydecanoic acid	189.1486	0.2	199.1820	-0.2	1	6.1E+01
**224**	C7H3NO3			150.0184	-1.1	157.0426	3.7	7	3.9E+02
**225**	C3H6N2O2	HMDB14405	Cycloserine	103.0505	2.7	106.0606	3.3	2	5.6E+03
**226**	C9H16O3	HMDB60287	4-Hydroperoxy-2-nonenal	173.1173	0.5	182.1472	-0.9	2	1.2E+07
**227**	C13H22O	YMDB01701	Geranyl acetone	195.1746	1.4	208.2177	-1.0	3	9.5E+04
**228**	C12H20O	HMDB31181	Homodihydrojasmone	181.1589	1.2	193.1985	-2.2	3	3.6E+05
**229**	C6H10O2	YMDB01685	Ethyl lactate	115.0753	-0.8	121.0955	0.3	2	3.3E+09
**230**	C5H10O2	YMDB01390	Propyl acetate	103.0756	2.1	108.0923	1.6	1	1.1E+07
**231**	C3H6O2	YMDB01684	Ethyl formate	75.0442	1.7	78.0543	2.5	1	2.7E+06
**232**	C5H8O	HMDB31407	Cyclopentanone	85.0651	3.2	90.0818	2.5	2	8.1E+05
**233**	C4H8O	YMDB01335	Ethoxy ethene	73.0649	1.8	77.0781	-1.1	1	Inf
**234**	C4H6O2	YMDB16203	But-2-enoic acid	87.0443	3.1	91.0575	0.6	2	3.9E+03
**235**	C11H14O2	HMDB31864	Methyleugenol	179.1066	-0.2	190.1436	0.3	5	2.0E+01
**236**	C15H24O3	HMDB35729	Dihydromyoporone	253.1804	2.1	268.2300	-0.1	4	2.7E+03
**237**	C11H18O2	HMDB35156	Neryl formate	183.1377	-1.2	194.1747	-0.7	3	Inf
**238**	C6H12O2	YMDB01424	Hexanoic acid	117.0913	2.2	123.1110	-0.5	1	4.3E+09
**239**	C2H4O2	YMDB00056	Acetic acid	61.0285	1.4	63.0351	-0.4	1	7.8E+10
**240**	C4H6O	HMDB61873	3-Buten-2-one	71.0494	3.2	75.0626	0.1	2	8.6E+05
**241**	C14H20			189.1638	0.0	203.2104	-1.4	5	Inf
**242**	C15H22O3	HMDB36563	Valerenolic acid	251.1648	2.5	266.2140	-1.4	5	1.0E+03

**243**	C15H24O4	HMDB36150	4,11,13,15-Tetrahydroridentin B	269.1749	0.5	284.2246	-1.5	4	6.8E+02
**244**	C5H12N2			101.1071	-2.7	106.1242	1.3	1	1.4E+05
**245**	C18H32O	HMDB37543	(±)-(Z)-2-(5-Tetradecenyl)cyclobutanone	265.2526	0.1	283.3129	-0.1	3	Inf
**246**	C11H14O4	HMDB13070	Sinapyl alcohol	211.0965	0.3	222.1330	-1.4	5	7.3E+00
**247**	C8H9NO4	HMDB00017	4-Pyridoxic acid	184.0606	1.0	192.0865	-3.6	5	3.5E+00
**248**	C16H31NO2			270.2434	2.2	286.2966	0.7	2	Inf
**249**	C4H9NO	HMDB31581	Morpholine	88.0756	-0.8	92.0893	1.8	1	1.6E+04
**250**	C14H22O	YMDB01634	alpha-Isomethyl-ionone	207.1743	-0.2	221.2214	0.5	4	2.5E+03
**251**	C13H16	YMDB01576	1,1,6-Trimethyl-1,2-dihydronaphthalene	173.1325	0.4	186.1756	-2.2	6	Inf
**252**	C8H8	YMDB16080	Styrene	105.0701	1.9	113.0964	-2.1	5	3.7E+04
**253**	C11H16O3	HMDB37725	Hexyl 2-furoate	197.1171	-0.4	208.1541	0.0	4	Inf
**254**	C15H22O4	HMDB30104	Humulinic acid A	267.1593	0.9	282.2086	-2.8	5	4.8E+02
**255**	C14H18			187.1482	0.6	201.1948	-0.9	6	9.4E+03
**256**	C11H18O3			199.1331	1.4	210.1696	-0.4	3	Inf
**257**	C15H26O4	HMDB40459	Ethylene brassylate	271.1904	0.2	286.2410	1.3	3	3.7E+02
**258**	C15H29NO4	HMDB00791	L-Octanoylcarnitine	288.2158	-4.0	303.2673	0.2	2	7.0E+02
**259**	C13H24O	HMDB41490	Dihydrogeranylacetone	197.1897	-1.4	210.2332	-1.4	2	Inf
**260**	C11H18O	HMDB36190	2-Heptylfuran	167.1432	1.1	178.1797	-1.0	3	Inf
**261**	C11H20O2	YMDB16108	gamma-Undecalactone	185.1537	0.7	196.1907	1.1	2	Inf
**262**	C13H24O2	HMDB37226	Citronellyl propionate	213.1851	1.0	226.2278	-3.1	2	2.2E+03
**263**	C17H32O	HMDB41335	8-Heptadecenal	253.2529	1.4	270.3097	0.4	2	Inf

*Matching formula with at least one compound of the yeast or human metabolome databases.

**Signal-to-noise ratio computed as the ratio of the variances of the signals of the experiment with yeast and the negative control where we spiked glucose into sterile medium (see example in Figure S1).

Table S2 Mass spectral features detected upon injecting $^{13}C_1$-glucose to WT, *zwf1* and *pfk1*. The order corresponds to the one shown in the heatmap from Figure 2d (top-to-bottom). The molecular *formulae* were generated assuming $[M+H]^+$ ion formation.

#	*m/z*	molecular formula (^{12}C & ^{13}C)	Unique molecular formula	Mass error (ppm)
**1**	269.0499	C11(13C)1H5N5O3	C12H5N5O3	0.04
**2**	270.0530	C10(13C)2H5N5O3	C12H5N5O3	-1.01
**3**	268.0464	C12H5N5O3	C12H5N5O3	-0.58
**4**	149.0870	C8(13C)2H10O	C10H10O	-0.95
**5**	138.1034	C3(13C)2H13NO3	C5H13NO3	-1.07
**6**	271.0457	C10(13C)3H5N3O4	C13H5N3O4	1.40
**7**	79.0663	C1(13C)2H8O2	C3H8O2	-1.07
**8**	148.0835	C9(13C)1H10O	C10H10O	-2.09
**9**	227.0386	C8(13C)1H7NO6	C9H7NO6	2.97
**10**	226.0356	C9H7NO6	C9H7NO6	4.24
**11**	228.0422	C7(13C)2H7NO6	C9H7NO6	3.69
**12**	228.0314	C5(13C)4H5NO6	C9H5NO6	-4.29
**13**	108.0197	-	-	-
**14**	209.0277	C8(13C)1H5NO5	C9H5NO5	1.32
**15**	77.0427	C1(13C)1H5NO2	C2H5NO2	0.71
**16**	215.1554	C10(13C)2H20O3	C12H20O3	0.92
**17**	192.0480	C8(13C)1H6N2O3	C9H6N2O3	-2.41
**18**	200.1640	C9(13C)3H20O2	C12H20O2	1.60
**19**	199.1605	C10(13C)2H20O2	C12H20O2	0.77
**20**	172.1326	C7(13C)3H16O2	C10H16O2	1.34
**21**	74.0317	C2(13C)1H4O2	C3H4O2	-0.79
**22**	176.0911	C5(13C)3H12O4	C8H12O4	1.33
**23**	170.0530	C7(13C)1H8O4	C8H8O4	0.55

**24**	168.0733	C8(13C)1H10O3	C9H10O3	-2.13
**25**	202.1155	C9(13C)1H16O4	C10H16O4	-0.06
**26**	109.0559	C5(13C)2H6O	C7H6O	0.73
**27**	102.0268	C3(13C)1H4O3	C4H4O3	1.26
**28**	192.1676	C9(13C)1H22O3	C10H22O3	0.54
**29**	191.1641	C10H22O3	C10H22O3	-0.33
**30**	201.1120	C10H16O4	C10H16O4	-0.89
**31**	160.0684	C6(13C)1H10O4	C7H10O4	-0.90
**32**	236.1129	C7(13C)3H16O6	C10H16O6	3.66
**33**	159.0653	C7H10O4	C7H10O4	0.88
**34**	167.0612	C7(13C)2H8O3	C9H8O3	-0.57
**35**	186.0842	C8(13C)1H12O4	C9H12O4	0.22
**36**	105.0459	C2(13C)2H6O3	C4H6O3	1.90
**37**	133.0405	C3(13C)2H6O4	C5H6O4	-0.49
**38**	103.0299	C2(13C)2H4O3	C4H4O3	-1.51
**39**	190.1337	C5(13C)5H16O3	C10H16O3	-1.53
**40**	201.1030	C8(13C)2H14O4	C10H14O4	-0.99
**41**	183.0925	C8(13C)2H12O3	C10H12O3	-0.80
**42**	71.0404	C2(13C)2H4O	C4H4O	2.90
**43**	168.1015	C7(13C)3H12O2	C10H12O2	2.53
**44**	154.0858	C6(13C)3H10O2	C9H10O2	2.47
**45**	131.0886	C3(13C)4H10O2	C7H10O2	-1.41
**46**	143.0887	C4(13C)4H10O2	C8H10O2	-0.31
**47**	107.0525	(13C)4H6O3	C4H6O3	0.77
**48**	126.0629	C6(13C)1H8O2	C7H8O2	-1.10
**49**	91.0391	C3H6O3	C3H6O3	1.93
**50**	148.0597	C3(13C)3H8O4	C6H8O4	0.99
**51**	113.0508	C4(13C)2H6O2	C6H6O2	-0.14
**52**	100.0475	C4(13C)1H6O2	C5H6O2	1.27
**53**	172.1048	C8(13C)1H14O3	C9H14O3	-0.59
**54**	138.0631	C7(13C)1H8O2	C8H8O2	0.02

**55**	104.0424	C3(13C)1H6O3	C4H6O3	0.31
**56**	154.0580	C7(13C)1H8O3	C8H8O3	0.31
**57**	76.0392	C2H5NO2	C2H5NO2	-1.49
**58**	171.0475	C4(13C)4H6O4	C8H6O4	1.41
**59**	163.0870	C9H10N2O	C9H10N2O	2.68
**60**	161.0710	C9H8N2O	C9H8N2O	0.52
**61**	70.0651	C4H7N	C4H7N	-0.27
**62**	188.1276	C7(13C)3H16O3	C10H16O3	1.47
**63**	187.1240	C8(13C)2H16O3	C10H16O3	0.59
**64**	186.1205	C9(13C)1H16O3	C10H16O3	-0.31
**65**	173.1083	C7(13C)2H14O3	C9H14O3	0.38
**66**	169.1135	C8(13C)2H14O2	C10H14O2	0.95
**67**	144.0734	C6(13C)1H10O3	C7H10O3	-1.32
**68**	131.0613	C4(13C)2H8O3	C6H8O3	-0.51
**69**	114.0632	C5(13C)1H8O2	C6H8O2	1.50
**70**	145.0770	C5(13C)2H10O3	C7H10O3	-0.16
**71**	115.0663	C4(13C)2H8O2	C6H8O2	-0.98
**72**	101.0506	C3(13C)2H6O2	C5H6O2	-1.56
**73**	89.0509	C2(13C)2H6O2	C4H6O2	1.73
**74**	219.0477	C8(13C)4H6O4	C12H6O4	1.61
**75**	133.0768	C4(13C)2H10O3	C6H10O3	-1.23
**76**	132.0737	C5(13C)1H10O3	C6H10O3	0.91
**77**	141.1184	C7(13C)2H14O	C9H14O	-0.18
**78**	129.0820	C5(13C)2H10O2	C7H10O2	-0.53
**79**	115.1026	C5(13C)2H12O	C7H12O	-1.83
**80**	147.0925	C5(13C)2H12O3	C7H12O3	-0.81
**81**	146.0894	C6(13C)1H12O3	C7H12O3	1.13
**82**	101.0873	C4(13C)2H10O	C6H10O	1.95
**83**	157.1134	C7(13C)2H14O2	C9H14O2	0.13
**84**	155.0978	C7(13C)2H12O2	C9H12O2	0.75
**85**	113.0870	C5(13C)2H10O	C7H10O	-1.01

**86**	99.0713	C4(13C)2H8O	C6H8O	-1.60
**87**	97.0558	C4(13C)2H6O	C6H6O	-0.64
**88**	156.0736	C7(13C)1H10O3	C8H10O3	-0.31
**89**	160.0962	C5(13C)3H12O3	C8H12O3	1.18
**90**	159.0927	C6(13C)2H12O3	C8H12O3	0.14
**91**	158.0891	C7(13C)1H12O3	C8H12O3	-0.92
**92**	146.0805	C4(13C)3H10O3	C7H10O3	0.99
**93**	142.0942	C7(13C)1H12O2	C8H12O2	-1.35
**94**	145.1043	C4(13C)4H12O2	C8H12O2	-0.97
**95**	144.1012	C5(13C)3H12O2	C8H12O2	0.99
**96**	147.1198	C4(13C)4H14O2	C8H14O2	-1.62
**97**	129.1093	C4(13C)4H12O	C8H12O	-1.45
**98**	162.1117	C5(13C)3H14O3	C8H14O3	0.57
**99**	161.1082	C6(13C)2H14O3	C8H14O3	-0.47
**100**	160.1051	C7(13C)1H14O3	C8H14O3	1.30
**101**	144.1102	C7(13C)1H14O2	C8H14O2	1.13
**102**	146.1168	C5(13C)3H14O2	C8H14O2	0.32
**103**	145.1132	C6(13C)2H14O2	C8H14O2	-0.83
**104**	127.1027	C6(13C)2H12O	C8H12O	-0.55
**105**	127.0664	C5(13C)2H8O2	C7H8O2	0.22
**106**	157.0771	C6(13C)2H10O3	C8H10O3	0.75
**107**	143.0977	C6(13C)2H12O2	C8H12O2	-0.17
**108**	141.0821	C6(13C)2H10O2	C8H10O2	0.51
**109**	125.0872	C6(13C)2H10O	C8H10O	0.22
**110**	85.0557	C3(13C)2H6O	C5H6O	-2.39
**111**	186.1120	C7(13C)3H14O3	C10H14O3	2.01
**112**	174.1119	C6(13C)3H14O3	C9H14O3	1.34
**113**	172.0963	C6(13C)3H12O3	C9H12O3	1.91
**114**	175.1235	C7(13C)2H16O3	C9H16O3	-2.75
**115**	167.0980	C8(13C)2H12O2	C10H12O2	1.54
**116**	151.1030	C8(13C)2H12O	C10H12O	1.40

**117**	187.0878	C7(13C)2H12O4	C9H12O4	1.11
**118**	185.1085	C8(13C)2H14O3	C10H14O3	1.12
**119**	148.0687	C5(13C)1H10O4	C6H10O4	1.12
**120**	162.0840	C6(13C)1H12O4	C7H12O4	-1.49
**121**	169.0772	C7(13C)2H10O3	C9H10O3	1.53
**122**	171.0928	C7(13C)2H12O3	C9H12O3	0.95
**123**	143.0614	C5(13C)2H8O3	C7H8O3	0.52
**124**	173.0721	C6(13C)2H10O4	C8H10O4	0.95
**125**	139.0666	C6(13C)2H8O2	C8H8O2	1.22
**126**	174.0756	C5(13C)3H10O4	C8H10O4	1.90
**127**	159.0564	C5(13C)2H8O4	C7H8O4	0.75
**128**	155.0611	C6(13C)2H8O3	C8H8O3	-1.52
**129**	141.0454	C5(13C)2H6O3	C7H6O3	-1.99
**130**	125.0509	C5(13C)2H6O2	C7H6O2	1.00
**131**	158.0528	C6(13C)1H8O4	C7H8O4	-0.30
**132**	142.0579	C6(13C)1H8O3	C7H8O3	-0.66
**133**	172.0685	C7(13C)1H10O4	C8H10O4	-0.02
**134**	130.0577	C5(13C)1H8O3	C6H8O3	-1.80
**135**	106.0579	C3(13C)1H8O3	C4H8O3	-0.61
**136**	120.0373	C3(13C)1H6O4	C4H6O4	0.65
**137**	88.0474	C3(13C)1H6O2	C4H6O2	-0.16
**138**	87.0349	C2(13C)2H4O2	C4H4O2	-2.32
**139**	134.0803	C3(13C)3H10O3	C6H10O3	0.03
**140**	118.0854	C3(13C)3H10O2	C6H10O2	-0.36
**141**	117.1096	C3(13C)4H12O	C7H12O	1.06
**142**	158.0811	C5(13C)3H10O3	C8H10O3	4.66
**143**	142.1219	C6(13C)3H14O	C9H14O	1.00
**144**	116.0425	C4(13C)1H6O3	C5H6O3	1.49
**145**	98.0683	C5(13C)1H8O	C6H8O	1.29
**146**	96.0527	C5(13C)1H6O	C6H6O	2.32
**147**	175.0876	C6(13C)2H12O4	C8H12O4	0.38

**148**	132.0648	C3(13C)3H8O3	C6H8O3	0.76
**149**	128.1058	C5(13C)3H12O	C8H12O	-2.76
**150**	140.0786	C7(13C)1H10O2	C8H10O2	-0.68
**151**	124.0837	C7(13C)1H10O	C8H10O	-1.13
**152**	84.0526	C4(13C)1H6O	C5H6O	0.97
**153**	125.0540	C4(13C)1H5N3O	C5H5N3O	1.07
**154**	135.0561	C3(13C)2H8O4	C5H8O4	-1.20
**155**	124.0505	C5H5N3O	C5H5N3O	-0.27
**156**	123.0474	(13C)4H6O4	C4H6O4	1.04
**157**	109.0595	C4(13C)1H5N3	C5H5N3	4.96
**158**	141.0490	C4(13C)1H5N3O2	C5H5N3O2	1.27
**159**	123.0385	C4(13C)1H3N3O	C5H3N3O	1.88
**160**	140.0455	C5H5N3O2	C5H5N3O2	0.09
**161**	122.0350	C5H3N3O	C5H3N3O	0.52
**162**	121.0319	(13C)4H4O4	C4H4O4	1.86
**163**	92.0422	C2(13C)1H6O3	C3H6O3	-1.19
**164**	108.0560	C5H5N3	C5H5N3	3.45
**165**	89.0420	(13C)4H4O2	C4H4O2	1.50
**166**	64.0476	C1(13C)1H6O2	C2H6O2	2.45
**167**	178.1703	C5(13C)5H20O2	C10H20O2	-0.44
**168**	177.1668	C6(13C)4H20O2	C10H20O2	-1.39
**169**	176.1637	C7(13C)3H20O2	C10H20O2	0.22
**170**	175.1606	C8(13C)2H20O2	C10H20O2	1.84
**171**	174.1571	C9(13C)1H20O2	C10H20O2	0.89
**172**	214.2254	C6(13C)9H24	C15H24	0.50
**173**	213.2219	C7(13C)8H24	C15H24	-0.28
**174**	155.1525	C5(13C)6H16	C11H16	-0.77
**175**	225.1947	C9(13C)6H22O	C15H22O	0.89
**176**	224.1916	C10(13C)5H22O	C15H22O	2.15
**177**	223.1881	C11(13C)4H22O	C15H22O	1.42
**178**	222.1846	C12(13C)3H22O	C15H22O	0.67

**179**	230.2293	C8(13C)7H26O	C15H26O	0.75
**180**	229.2262	C9(13C)6H26O	C15H26O	1.99
**181**	228.2227	C10(13C)5H26O	C15H26O	1.27
**182**	168.1647	C7(13C)5H18	C12H18	-1.44
**183**	227.2102	C9(13C)6H24O	C15H24O	0.45
**184**	226.2067	C10(13C)5H24O	C15H24O	-0.28
**185**	209.2002	C9(13C)6H22	C15H22	2.89
**186**	225.2036	C11(13C)4H24O	C15H24O	0.97
**187**	208.1966	C10(13C)5H22	C15H22	2.10
**188**	227.2192	C11(13C)4H26O	C15H26O	0.54
**189**	140.1337	C5(13C)5H14	C10H14	0.86
**190**	127.1301	C5(13C)4H14	C9H14	-1.48
**191**	126.1270	C6(13C)3H14	C9H14	0.76
**192**	113.1144	C4(13C)4H12	C8H12	-2.06
**193**	225.2121	C13(13C)2H26O	C15H26O	-0.94
**194**	125.1235	C7(13C)2H14	C9H14	-0.57
**195**	112.1113	C5(13C)3H12	C8H12	0.46
**196**	111.1078	C6(13C)2H12	C8H12	-1.04
**197**	212.2188	C8(13C)7H24	C15H24	1.06
**198**	154.1494	C6(13C)5H16	C11H16	1.07
**199**	211.2153	C9(13C)6H24	C15H24	0.27
**200**	153.1459	C7(13C)4H16	C11H16	-0.02
**201**	126.1180	C4(13C)5H12	C9H12	0.60
**202**	210.2122	C10(13C)5H24	C15H24	1.62
**203**	125.1145	C5(13C)4H12	C9H12	-0.73
**204**	124.1114	C6(13C)3H12	C9H12	1.55
**205**	111.0988	C4(13C)4H10	C8H10	-1.22
**206**	128.1336	C4(13C)5H14	C9H14	-0.16
**207**	210.2041	C10(13C)4H23N	C14H23N	1.82
**208**	228.2137	C8(13C)7H24O	C15H24O	1.18
**209**	114.1179	C3(13C)5H12	C8H12	-0.57

**210**	85.0834	C2(13C)4H8	C6H8	1.54
**211**	151.1303	C7(13C)4H14	C11H14	0.62
**212**	149.1237	C9(13C)2H14	C11H14	1.41
**213**	121.0924	C7(13C)2H10	C9H10	1.01
**214**	150.1358	C10(13C)1H16	C11H16	-0.35
**215**	122.1044	C8(13C)1H12	C9H12	-1.16
**216**	97.0831	C3(13C)4H8	C7H8	-1.86
**217**	110.0957	C5(13C)3H10	C8H10	1.35
**218**	96.0800	C4(13C)3H8	C7H8	1.09
**219**	208.2051	C12(13C)3H24	C15H24	0.03
**220**	207.2016	C13(13C)2H24	C15H24	-0.77
**221**	152.1424	C8(13C)3H16	C11H16	-1.12
**222**	151.1393	C9(13C)2H16	C11H16	0.75
**223**	209.2087	C11(13C)4H24	C15H24	0.83
**224**	123.1079	C7(13C)2H12	C9H12	0.21
**225**	223.1970	C13(13C)2H24O	C15H24O	1.50
**226**	206.1896	C12(13C)3H22	C15H22	0.50
**227**	205.1865	C13(13C)2H22	C15H22	1.89
**228**	224.2001	C12(13C)3H24O	C15H24O	0.23
**229**	207.1927	C11(13C)4H22	C15H22	-0.87
**230**	109.0922	C6(13C)2H10	C8H10	-0.17
**231**	95.0765	C5(13C)2H8	C7H8	-0.67
**232**	124.1204	C8(13C)1H14	C9H14	1.72
**233**	110.1047	C7(13C)1H12	C8H12	1.53
**234**	136.1201	C9(13C)1H14	C10H14	-0.72
**235**	96.0890	C6(13C)1H10	C7H10	1.30
**236**	82.0733	C5(13C)1H8	C6H8	0.98
**237**	138.1477	C4(13C)3H18O2	C7H18O2	-2.09
**238**	137.1447	C5(13C)2H18O2	C7H18O2	-0.03
**239**	136.1416	C6(13C)1H18O2	C7H18O2	2.06
**240**	93.0610	C5(13C)2H6	C7H6	0.36

**241**	134.0530	C4(13C)1H8O4	C5H8O4	0.91
**242**	119.0531	C3(13C)1H7NO3	C4H7NO3	-1.39
**243**	74.0958	C2(13C)3H10	C5H10	2.40
**244**	73.0922	C3(13C)2H10	C5H10	0.13
**245**	72.0887	C4(13C)1H10	C5H10	-2.20
**246**	218.1745	C9(13C)3H22O3	C12H22O3	1.23
**247**	217.1710	C10(13C)2H22O3	C12H22O3	0.47
**248**	167.1616	C8(13C)4H18	C12H18	0.25
**249**	166.1581	C9(13C)3H18	C12H18	-0.76
**250**	165.1550	C10(13C)2H18	C12H18	0.96
**251**	149.1354	C4(13C)4H16O2	C8H16O2	-2.24
**252**	148.1323	C5(13C)3H16O2	C8H16O2	-0.34
**253**	147.1288	C6(13C)2H16O2	C8H16O2	-1.48
**254**	146.1257	C7(13C)1H16O2	C8H16O2	0.45
**255**	139.1302	C6(13C)4H14	C10H14	-0.34
**256**	99.0991	C3(13C)4H10	C7H10	1.77
**257**	138.1267	C7(13C)3H14	C10H14	-1.55
**258**	98.0956	C4(13C)3H10	C7H10	0.08
**259**	97.0921	C5(13C)2H10	C7H10	-1.65
**260**	84.0799	C3(13C)3H8	C6H8	-0.44
**261**	137.1236	C8(13C)2H14	C10H14	0.51
**262**	83.0768	C4(13C)2H8	C6H8	2.99
**263**	129.1183	C6(13C)2H14O	C8H14O	-1.29
**264**	81.0608	C4(13C)2H6	C6H6	-1.33
**265**	142.1493	C5(13C)5H16	C10H16	0.16
**266**	141.1458	C6(13C)4H16	C10H16	-1.02
**267**	140.1427	C7(13C)3H16	C10H16	1.00
**268**	139.1392	C8(13C)2H16	C10H16	-0.19
**269**	135.1081	C8(13C)2H12	C10H12	1.23
**270**	69.0611	C3(13C)2H6	C5H6	2.96
**271**	68.0576	C4(13C)1H6	C5H6	0.53

**272**	130.1223	C5(13C)3H14O	C8H14O	3.47
**273**	108.0887	C7(13C)1H10	C8H10	-1.72
**274**	226.2161	C12(13C)3H26O	C15H26O	1.80
**275**	173.1536	C10H20O2	C10H20O2	-0.07
**276**	94.0735	C6(13C)1H8	C7H8	2.36
**277**	158.1169	C6(13C)3H14O2	C9H14O2	1.18
**278**	164.1515	C11(13C)1H18	C12H18	-0.05
**279**	120.0888	C8(13C)1H10	C9H10	-0.37
**280**	224.2091	C14(13C)1H26O	C15H26O	0.32
**281**	80.0578	C5(13C)1H6	C6H6	2.22
**282**	256.1900	C12(13C)3H24O3	C15H24O3	0.56
**283**	78.0418	C5(13C)1H4	C6H4	-2.29
**284**	145.1222	C8H16O2	C8H16O2	-0.69
**285**	221.1810	C13(13C)2H22O	C15H22O	-0.08
**286**	137.1146	C6(13C)4H12	C10H12	0.36
**287**	135.1381	C7H18O2	C7H18O2	0.83
**288**	206.1806	C10(13C)5H20	C15H20	0.41
**289**	205.1771	C11(13C)4H20	C15H20	-0.41
**290**	220.1686	C12(13C)3H20O	C15H20O	-0.93
**291**	222.1756	C10(13C)5H20O	C15H20O	0.58
**292**	221.1725	C11(13C)4H20O	C15H20O	1.86
**293**	204.1740	C12(13C)3H20	C15H20	0.98
**294**	131.1253	C4(13C)4H14O	C8H14O	1.28
**295**	123.0990	C5(13C)4H10	C9H10	0.04
**296**	156.0933	C6(13C)2H11NO2	C8H11NO2	2.09
**297**	155.0898	C7(13C)1H11NO2	C8H11NO2	1.03
**298**	153.0823	C7(13C)2H10O2	C9H10O2	1.39
**299**	137.0873	C7(13C)2H10O	C9H10O	1.23
**300**	139.1029	C7(13C)2H12O	C9H12O	0.51
**301**	111.0715	C5(13C)2H8O	C7H8O	-0.16
**302**	150.1183	C6(13C)5H12	C11H12	2.38

**303**	221.1631	C9(13C)6H18O	C15H18O	-0.26
**304**	180.1652	C8(13C)5H18	C13H18	1.94
**305**	236.1635	C12(13C)3H20O2	C15H20O2	-0.67
**306**	218.1530	C12(13C)3H18O	C15H18O	-0.49
**307**	169.1409	C7(13C)4H16O	C11H16O	0.25
**308**	219.1655	C13(13C)2H20O	C15H20O	0.36
**309**	161.1234	C10(13C)2H14	C12H14	-0.62
**310**	160.1114	C9(13C)3H12	C12H12	1.03
**311**	129.1371	C3(13C)6H14	C9H14	1.13
**312**	167.1343	C9(13C)2H16O	C11H16O	0.95
**313**	179.1613	C9(13C)4H18	C13H18	-1.50
**314**	225.2319	C10(13C)6H26	C16H26	4.45
**315**	178.1587	C10(13C)3H18	C13H18	2.61
**316**	219.1565	C11(13C)4H18O	C15H18O	0.27
**317**	152.1339	C6(13C)5H14	C11H14	1.72
**318**	220.1600	C10(13C)5H18O	C15H18O	1.02
**319**	205.1682	C9(13C)6H18	C15H18	-0.50
**320**	141.1368	C4(13C)6H14	C10H14	-1.16
**321**	127.1215	C3(13C)6H12	C9H12	1.91
**322**	166.1491	C7(13C)5H16	C12H16	-0.88
**323**	112.1023	C3(13C)5H10	C8H10	0.28
**324**	203.1620	C11(13C)4H18	C15H18	2.28
**325**	198.1484	C9(13C)3H18O2	C12O18O2	2.10
**326**	138.0908	C6(13C)3H10O	C9H10O	2.43
**327**	136.1120	C9H13N	C9H13N	-0.41
**328**	150.1273	C8(13C)3H14	C11H14	2.52
**329**	122.0959	C6(13C)3H10	C9H10	2.37
**330**	238.1795	C12(13C)3H22O2	C15H22O2	0.82
**331**	183.1565	C8(13C)4H18O	C12H18O	0.48
**332**	181.1500	C10(13C)2H18O	C12H18O	1.12
**333**	226.2349	C9(13C)7H26	C16H26	3.17

**334**	163.1390	C10(13C)2H16	C12H16	-1.20
**335**	207.1842	C9(13C)6H20	C15H20	1.21
**336**	204.1651	C10(13C)5H18	C15H18	0.88
**337**	148.1113	C8(13C)3H12	C11H12	0.16
**338**	223.1791	C9(13C)6H20O	C15H20O	1.33
**339**	147.1077	C9(13C)2H12	C11H12	-0.98
**340**	203.1705	C13(13C)2H20	C15H20	0.16
**341**	202.1585	C12(13C)3H18	C15H18	1.47
**342**	139.0939	C5(13C)4H10O	C9H10O	0.37
**343**	211.1884	C3(13C)12H18	C152H18	-0.01
**344**	143.1528	C4(13C)6H16	C10H16	1.33
**345**	181.1773	C9(13C)4H20	C13H20	0.47
**346**	180.1742	C10(13C)3H20	C13H20	2.05
**347**	226.1977	C8(13C)7H22O	C15H22O	-0.37
**348**	179.1738	C4(13C)6H20O2	C10H20O2	0.49
**349**	169.1682	C6(13C)6H18	C12H18	-0.44
**350**	100.1022	C2(13C)5H10	C7H10	-1.10
**351**	214.1434	C2(13C)11H14O2	C131H14O2	-0.78
**352**	182.1808	C8(13C)5H20	C13H20	1.39
**353**	183.1292	C9(13C)2H16O2	C11H16O2	1.12
**354**	224.1826	C1(13C)15H16	C16H16	-0.74
**355**	184.1601	C7(13C)5H18O	C12H18O	1.38
**356**	181.1410	C8(13C)4H16O	C12H16O	1.01
**357**	197.1449	C10(13C)2H18O2	C12H18O2	1.26
**358**	138.1182	C5(13C)5H12	C10H12	1.57
**359**	124.1325	C3(13C)3H16O2	C6H16O2	0.96
**360**	121.1224	C6H16O2	C6H16O2	0.57
**361**	127.0938	C4(13C)4H10O	C8H10O	-0.71
**362**	108.1008	C2(13C)3H12O2	C5H12O2	-2.60
**363**	123.1290	C4(13C)2H16O2	C6H16O2	-0.39
**364**	122.1259	C5(13C)1H16O2	C6H16O2	1.93

**365**	59.0766	C2(13C)2H8	C4H8	-0.60
**366**	58.0735	C3(13C)1H8	C4H8	4.34
**367**	155.1614	C7(13C)4H18	C11H18	-0.64
**368**	212.2273	C10(13C)5H26	C15H26	-0.97
**369**	154.1579	C8(13C)3H18	C11H18	-1.73
**370**	232.2453	C8(13C)7H28O	C15H28O	2.27
**371**	211.2229	C3(13C)6H24N4O	C9H24N4O	2.20
**372**	130.1491	C4(13C)5H16	C9H16	-0.90
**373**	229.2347	C11(13C)4H28O	C15H28O	0.12
**374**	231.2413	C9(13C)6H28O	C15H28O	-0.39
**375**	230.2378	C10(13C)5H28O	C15H28O	-1.12
**376**	115.1304	C4(13C)4H14	C8H14	1.06
**377**	242.2106	C12(13C)3H26O2	C15H26O2	0.01
**378**	227.2281	C13(13C)2H28O	C15H28O	0.63
**379**	129.1461	C5(13C)4H16	C9H16	1.29
**380**	128.1425	C6(13C)3H16	C9H16	-0.01
**381**	114.1268	C5(13C)3H14	C8H14	-0.40
**382**	113.1233	C6(13C)2H14	C8H14	-1.88
**383**	76.0750	C1(13C)3H8O	C4H8O	2.35
**384**	73.0833	C1(13C)4H8	C5H8	-0.15
**385**	210.0397	C9H7NO5	C9H7NO5	0.06
**386**	195.0483	C8(13C)1H7NO4	C9H7NO4	0.69
**387**	244.2100	C10(13C)4H25NO2	C14H25NO2	3.39
**388**	196.1960	C9(13C)5H22	C14H22	-0.78
**389**	195.1930	C10(13C)4H22	C14H22	0.66
**390**	194.1895	C11(13C)3H22	C14H22	-0.19
**391**	87.0990	C2(13C)4H10	C6H10	0.39
**392**	242.2017	C10(13C)5H24O2	C15H24O2	-0.08
**393**	240.1951	C12(13C)3H24O2	C15H24O2	0.41
**394**	239.1915	C13(13C)2H24O2	C15H24O2	-0.29
**395**	127.1390	C7(13C)2H16	C9H16	-1.32

**396**	86.0955	C3(13C)3H10	C6H10	-1.56
**397**	100.1112	C4(13C)3H12	C7H12	-0.89
**398**	99.1081	C5(13C)2H12	C7H12	1.97
**399**	101.1147	C3(13C)4H12	C7H12	0.77
**400**	85.0924	C4(13C)2H10	C6H10	1.78
**401**	61.0558	C1(13C)2H6O	C3H6O	-0.55
**402**	212.0557	C9H9NO5	C9H9NO5	1.73
**403**	205.1977	C8(13C)4H24O2	C12H24O2	-2.96
**404**	238.0429	C10(13C)1H8O6	C11H8O6	0.66
**405**	213.0588	C8(13C)1H9NO5	C9H9NO5	0.39
**406**	197.0638	C8(13C)1H9NO4	C9H9NO4	0.19
**407**	169.0687	C7(13C)1H9NO3	C8H9NO3	-0.88
**408**	196.0603	C9H9NO4	C9H9NO4	-0.66
**409**	168.0657	C8H9NO3	C8H9NO3	0.80
**410**	234.2331	C9(13C)5H28O2	C14H28O2	0.42
**411**	233.2296	C10(13C)4H28O2	C14H28O2	-0.29
**412**	232.2265	C11(13C)3H28O2	C14H28O2	0.92
**413**	231.2230	C12(13C)2H28O2	C14H28O2	0.21
**414**	206.2012	C7(13C)5H24O2	C12H24O2	-2.14
**415**	204.1951	C9(13C)3H24O2	C12H24O2	0.62
**416**	203.1916	C10(13C)2H24O2	C12H24O2	-0.20
**417**	202.1881	C11(13C)1H24O2	C12H24O2	-1.03
**418**	75.0988	C1(13C)4H10	C5H10	-1.44
**419**	78.0906	C1(13C)3H10O	C4H10O	1.05
**420**	96.1011	C1(13C)3H12O2	C4H12O2	0.32
**421**	95.0976	C2(13C)2H12O2	C4H12O2	-1.44
**422**	77.0871	C2(13C)2H10O	C4H10O	-1.12
**423**	93.0910	C4H12O2	C4H12O2	-0.22
**424**	94.0945	C3(13C)1H12O2	C4H12O2	1.57
**425**	76.0840	C3(13C)1H10O	C4H10O	2.62
**426**	75.0805	C4H10O	C4H10O	0.41

**427**	109.1133	C3(13C)2H14O2	C5H14O2	-0.85
**428**	108.1102	C4(13C)1H14O2	C5H14O2	1.77
**429**	107.1067	C5H14O2	C5H14O2	0.23
**430**	62.0683	C2(13C)1H8O	C3H8O	2.50
**431**	61.0648	C3H8O	C3H8O	-0.22
**432**	61.0478	C1(13C)1H5NO	C2H5NO	0.15
**433**	371.1017	C20(13C)3H13NO4	C23H13NO4	-0.28
**434**	194.0448	C9H7NO4	C9H7NO4	-0.17
**435**	90.0907	C2(13C)3H10O	C5H10O	2.48
**436**	72.0802	C2(13C)3H8	C5H8	3.82
**437**	71.0767	C3(13C)2H8	C5H8	1.50
**438**	70.0732	C4(13C)1H8	C5H8	-0.88
**439**	237.0394	C11H8O6	C11H8O6	-0.04
**440**	60.0810	C3H9N	C3H9N	3.27
**441**	229.0345	C9H8O7	C9H8O7	0.89
**442**	123.0716	C6(13C)2H8O	C8H8O	1.01
**443**	170.1256	C9(13C)1H16O2	C10H16O2	-0.60
**444**	103.0751	C5H10O2	C5H10O2	-2.27
**445**	106.0852	C2(13C)3H10O2	C5H10O2	-1.73
**446**	105.0822	C3(13C)2H10O2	C5H10O2	0.96
**447**	104.0786	C4(13C)1H10O2	C5H10O2	-0.64
**448**	77.0508	C1(13C)2H6O2	C3H6O2	0.16
**449**	134.1166	C4(13C)3H14O2	C7H14O2	-0.71
**450**	133.1136	C5(13C)2H14O2	C7H14O2	1.42
**451**	132.1100	C6(13C)1H14O2	C7H14O2	0.17
**452**	118.0581	C4(13C)1H8O3	C5H8O3	0.65
**453**	120.1009	C3(13C)3H12O2	C6H12O2	-1.16
**454**	119.0979	C4(13C)2H12O2	C6H12O2	1.22
**455**	118.0943	C5(13C)1H12O2	C6H12O2	-0.19
**456**	117.0456	C3(13C)2H6O3	C5H6O3	-0.95
**457**	119.0616	C3(13C)2H8O3	C5H8O3	2.05

**458**	87.0717	C3(13C)2H8O	C5H8O	1.75
**459**	86.0681	C4(13C)1H8O	C5H8O	-0.18
**460**	190.1431	C7(13C)3H18O3	C10H18O3	0.95
**461**	189.1396	C8(13C)2H18O3	C10H18O3	0.07
**462**	188.1361	C9(13C)1H18O3	C10H18O3	-0.82
**463**	158.1442	C5(13C)5H16O	C10H16O	0.44
**464**	176.1547	C5(13C)5H18O2	C10H18O2	0.10
**465**	175.1512	C6(13C)4H18O2	C10H18O2	-0.85
**466**	157.1407	C6(13C)4H16O	C10H16O	-0.62
**467**	156.1376	C7(13C)3H16O	C10H16O	1.19
**468**	172.1411	C9(13C)1H18O2	C10H18O2	-1.16
**469**	171.1291	C8(13C)2H16O2	C10H16O2	0.38
**470**	173.1446	C8(13C)2H18O2	C10H18O2	-0.19
**471**	174.1482	C7(13C)3H18O2	C10H18O2	0.77
**472**	155.1341	C8(13C)2H16O	C10H16O	0.12
**473**	59.0403	C1(13C)2H4O	C3H4O	1.08
**474**	211.1973	C5(13C)10H20	C150H20	0.08
**475**	192.1587	C7(13C)3H20O3	C10H20O3	0.44
**476**	191.1556	C8(13C)2H20O3	C10H20O3	1.92
**477**	137.0999	C4(13C)1H13NO3	C5H13NO3	-2.30
**478**	122.0802	C2(13C)3H10O3	C5H10O3	-1.13
**479**	104.0697	C2(13C)3H8O2	C5H8O2	-0.83
**480**	101.0596	C5H8O2	C5H8O2	-1.36
**481**	119.0701	C5H10O3	C5H10O3	-1.58
**482**	120.0736	C4(13C)1H10O3	C5H10O3	-0.17
**483**	121.0771	C3(13C)2H10O3	C5H10O3	1.21
**484**	103.0666	C3(13C)2H8O2	C5H8O2	1.92
**485**	102.0631	C4(13C)1H8O2	C5H8O2	0.30
**486**	78.0633	C2(13C)1H8O2	C3H8O2	2.57
**487**	212.2004	C11(13C)3H24O	C14H24O	1.71
**488**	232.2175	C9(13C)5H26O2	C14H26O2	0.84

**489**	204.1852	C7(13C)5H22O2	C12H22O2	-3.89
**490**	230.2109	C11(13C)3H26O2	C14H26O2	1.35
**491**	229.2074	C12(13C)2H26O2	C14H26O2	0.63
**492**	202.1795	C9(13C)3H22O2	C12H22O2	1.10
**493**	201.1760	C10(13C)2H22O2	C12H22O2	0.28
**494**	132.1011	C4(13C)3H12O2	C7H12O2	0.02
**495**	93.0820	C2(13C)2H10O2	C4H10O2	-0.43
**496**	92.0790	C3(13C)1H10O2	C4H10O2	2.66
**497**	91.0754	C4H10O2	C4H10O2	0.84
**498**	89.0872	C3(13C)2H10O	C5H10O	0.62
**499**	88.0837	C4(13C)1H10O	C5H10O	-1.28
**500**	77.0597	C3H8O2	C3H8O2	0.42
**501**	60.0527	C2(13C)1H6O	C3H6O	4.21
**502**	107.0977	C3(13C)2H12O2	C5H12O2	0.04
**503**	106.0942	C4(13C)1H12O2	C5H12O2	-1.54
**504**	105.0911	C5H12O2	C5H12O2	1.15
**505**	230.0380	C7(13C)4H3N3O3	C11H3N3O3	-0.59
**506**	75.0715	C2(13C)2H8O	C4H8O	0.15
**507**	74.0680	C3(13C)1H8O	C4H8O	-2.12
**508**	57.0610	C2(13C)2H6	C4H6	1.10
**509**	56.0575	C3(13C)1H6	C4H6	-1.90
**510**	160.1325	C6(13C)3H16O2	C9H16O2	0.57
**511**	159.1289	C7(13C)2H16O2	C9H16O2	-0.48
**512**	143.1340	C7(13C)2H16O	C9H16O	-0.85
**513**	153.1186	C8(13C)2H14O	C10H14O	0.75
**514**	117.0819	C4(13C)2H10O2	C6H10O2	-1.79
**515**	185.1721	C8(13C)4H20O	C12H20O	-0.05
**516**	184.1690	C9(13C)3H20O	C12H20O	1.49
**517**	183.1655	C10(13C)2H20O	C12H20O	0.58
**518**	130.0945	C6(13C)1H12O2	C7H12O2	0.91
**519**	131.0976	C5(13C)2H12O2	C7H12O2	-1.26

**520**	116.0788	C5(13C)1H10O2	C6H10O2	0.64
**521**	186.1756	C7(13C)5H20O	C12H20O	0.85
**522**	163.1238	C6(13C)2H16O3	C8H16O3	-1.05
**523**	149.1081	C5(13C)2H14O3	C7H14O3	-1.45
**524**	148.1050	C6(13C)1H14O3	C7H14O3	0.46
**525**	213.2035	C10(13C)4H24O	C14H24O	0.37
**526**	135.0928	C4(13C)2H12O3	C6H12O3	1.41
**527**	134.0893	C5(13C)1H12O3	C6H12O3	0.18
**528**	107.0897	C3(13C)1H11NO2	C4H11NO2	0.43
**529**	158.1254	C8(13C)1H16O2	C9H16O2	-1.54
**530**	76.0473	C2(13C)1H6O2	C3H6O2	-2.05
**531**	89.0599	C4H8O2	C4H8O2	1.96
**532**	92.0700	C1(13C)3H8O2	C4H8O2	2.44
**533**	91.0665	C2(13C)2H8O2	C4H8O2	0.62
**534**	90.0630	C3(13C)1H8O2	C4H8O2	-1.23
**535**	73.0560	C2(13C)2H6O	C4H6O	1.48
**536**	72.0524	C3(13C)1H6O	C4H6O	-0.83
**537**	141.1104	C7(13C)1H13NO	C8H13NO	0.12
**538**	107.0767	C6(13C)2H8	C8H8	0.73
**539**	106.0731	C7(13C)1H8	C8H8	-0.84
**540**	62.0347	H3N3O	H3N3O	-3.10
**541**	108.0735	C3(13C)1H10O3	C4H10O3	-1.50
**542**	63.0351	(13C)2H4O2	C2H4O2	-0.51
**543**	62.0320	C1(13C)1H4O2	C2H4O2	4.10
**544**	61.0285	C2H4O2	C2H4O2	1.40
**545**	105.0611	C6(13C)2H6	C8H6	1.67
**546**	164.1273	C5(13C)3H16O3	C8H16O3	-0.03
**547**	150.1116	C4(13C)3H14O3	C7H14O3	-0.33
**548**	145.1410	C5(13C)4H16O	C9H16O	1.46
**549**	142.1139	C6(13C)2H13NO	C8H13NO	1.29
**550**	104.0576	C7(13C)1H6	C8H6	0.07

**551**	188.1643	C8(13C)3H20O2	C11H20O2	3.34
**552**	109.0765	C2(13C)2H10O3	C4H10O3	-4.09
**553**	71.0494	C4H6O	C4H6O	3.18
**554**	162.1207	C7(13C)1H16O3	C8H16O3	0.69
**555**	152.1150	C9(13C)1H14O	C10H14O	-0.34
**556**	105.0701	C8H8	C8H8	1.86
**557**	182.1620	C11(13C)1H20O	C12H20O	-0.33
**558**	56.0212	C2(13C)1H2O	C3H2O	-0.13
**559**	262.2560	C9(13C)7H30O2	C16H30O2	2.35
**560**	288.2803	C13(13C)5H34O2	C18H34O2	1.29
**561**	242.2384	C11(13C)5H28O	C16H28O	1.38
**562**	258.2419	C13(13C)3H30O2	C16H30O2	-0.20
**563**	257.2388	C14(13C)2H30O2	C16H30O2	0.90
**564**	241.2349	C12(13C)4H28O	C16H28O	0.69
**565**	260.2489	C11(13C)5H30O2	C16H30O2	1.09
**566**	259.2463	C5(13C)12H26O	C17H26O	1.50
**567**	261.2529	C3(13C)14H26O	C17H26O	1.04
**568**	256.2263	C13(13C)3H28O2	C16H28O2	0.18
**569**	255.2237	C7(13C)10H24O	C17H24O	0.59
**570**	187.1603	C9(13C)2H20O2	C11H20O2	0.06
**571**	216.1952	C10(13C)3H24O2	C13H24O2	1.24
**572**	201.2038	C9(13C)4H24O	C13H24O	1.93
**573**	239.2193	C12(13C)4H26O	C16H26O	1.10
**574**	262.2918	C10(13C)7H34O	C17H34O	0.26
**575**	257.2746	C15(13C)2H34O	C17H34O	-1.22
**576**	202.2069	C8(13C)5H24O	C13H24O	0.52
**577**	199.1972	C4(13C)10H20	C14H20	-0.62
**578**	197.1996	C8(13C)6H22	C14H22	0.07
**579**	198.2031	C7(13C)7H22	C14H22	0.91
**580**	203.2104	C7(13C)6H24O	C13H24O	1.34
**581**	182.1898	C10(13C)3H22	C13H22	1.50

**582**	183.1928	C9(13C)4H22	C13H22	-0.06
**583**	215.1917	C11(13C)2H24O2	C13H24O2	0.47
**584**	171.1654	C9(13C)2H20O	C11H20O	-0.20
**585**	214.2070	C9(13C)5H24O	C14H24O	1.15
**586**	241.2071	C13(13C)2H26O2	C15H26O2	-0.68
**587**	193.1864	C12(13C)2H22	C14H22	1.28
**588**	117.0371	C1(13C)4H4O3	C5H4O3	2.74
**589**	177.1395	C7(13C)2H18O3	C9H18O3	-0.72
**590**	178.1430	C6(13C)3H18O3	C9H18O3	0.22
**591**	176.1364	C8(13C)1H18O3	C9H18O3	0.89
**592**	108.0932	C2(13C)2H11NO2	C4H11NO2	1.98
**593**	245.2118	C7(13C)8H24O2	C15H24O2	0.13
**594**	244.2168	C10(13C)5H26O2	C15H26O2	-2.31
**595**	126.1359	C8(13C)1H16	C9H16	0.92
**596**	84.0889	C5(13C)1H10	C6H10	-0.20
**597**	228.2402	C14(13C)1H30O	C15H30O	-0.53
**598**	98.1046	C6(13C)1H12	C7H12	0.28
**599**	58.0367	C2(13C)1H4O	C3H4O	-1.81
**600**	260.2762	C10(13C)7H32O	C17H32O	0.63
**601**	246.2328	C10(13C)5H28O2	C15H28O2	-0.86
**602**	243.2231	C13(13C)2H28O2	C15H28O2	0.77
**603**	177.1946	C5(13C)6H22O	C11H22O	0.49
**604**	260.2852	C12(13C)5H34O	C17H34O	0.71
**605**	259.2817	C13(13C)4H34O	C17H34O	0.07
**606**	261.2887	C11(13C)6H34O	C17H34O	1.35
**607**	258.2782	C14(13C)3H34O	C17H34O	-0.57
**608**	148.1596	C4(13C)5H18O	C9H18O	-1.14
**609**	147.1566	C5(13C)4H18O	C9H18O	0.78
**610**	146.1531	C6(13C)3H18O	C9H18O	-0.35
**611**	145.1495	C7(13C)2H18O	C9H18O	-1.51
**612**	87.1079	C4(13C)2H12	C6H12	0.62

**613**	172.1778	C10(13C)1H22O	C11H22O	0.89
**614**	144.1465	C8(13C)1H18O	C9H18O	0.45
**615**	116.1151	C6(13C)1H14O	C7H14O	-0.20
**616**	204.2229	C8(13C)5H26O	C13H26O	2.25
**617**	203.2194	C9(13C)4H26O	C13H26O	1.44
**618**	202.2154	C10(13C)3H26O	C13H26O	-1.61
**619**	201.2123	C11(13C)2H26O	C13H26O	-0.21
**620**	255.2591	C15(13C)2H32O	C17H32O	-0.86
**621**	205.2259	C7(13C)6H26O	C13H26O	0.85
**622**	176.1915	C6(13C)5H22O	C11H22O	2.10
**623**	175.1880	C7(13C)4H22O	C11H22O	1.16
**624**	174.1844	C8(13C)3H22O	C11H22O	0.21
**625**	173.1809	C9(13C)2H22O	C11H22O	-0.75
**626**	259.2732	C11(13C)6H32O	C17H32O	1.73
**627**	258.2701	C12(13C)5H32O	C17H32O	2.83
**628**	234.2609	C8(13C)7H30O	C15H30O	1.84
**629**	119.1252	C3(13C)4H14O	C7H14O	0.23
**630**	118.1217	C4(13C)3H14O	C7H14O	-1.19
**631**	117.1186	C5(13C)2H14O	C7H14O	1.23
**632**	233.2569	C9(13C)6H30O	C15H30O	-0.80
**633**	229.2437	C13(13C)2H30O	C15H30O	0.20
**634**	232.2538	C10(13C)5H30O	C15H30O	0.42
**635**	231.2503	C11(13C)4H30O	C15H30O	-0.30
**636**	230.2472	C12(13C)3H30O	C15H30O	0.93

Table S3. Compounds contributing to PC1 (*i.e.*, absolute value loading > 0.01)

#	*m/z*	molecular formula (^{12}C & ^{13}C)	Unique molecular formula	ID	Loading value
**1**	159.0653241	C7H10O4	C7H10O4		-0.02
**2**	145.1132461	C6(13C)2H14O2	C8H14O2		0.01
**3**	178.1703032	C5(13C)5H20O2	C10H20O2	Ethyl octanoate	0.01
**3**	177.1667822	C6(13C)4H20O2	C10H20O2	Ethyl octanoate	0.06
**3**	176.1637092	C7(13C)3H20O2	C10H20O2	Ethyl octanoate	0.12
**3**	175.1606362	C8(13C)2H20O2	C10H20O2	Ethyl octanoate	0.13
**3**	174.1571152	C9(13C)1H20O2	C10H20O2	Ethyl octanoate	0.07
**4**	112.1112931	C5(13C)3H12	C8H12		0.01
**4**	111.1077721	C6(13C)2H12	C8H12		0.01
**5**	212.2187852	C8(13C)7H24	C15H24	Farnesene	0.03
**5**	211.2152642	C9(13C)6H24	C15H24	Farnesene	0.07
**5**	210.2121912	C10(13C)5H24	C15H24	Farnesene	0.11
**5**	208.2051492	C12(13C)3H24	C15H24	Farnesene	0.08
**5**	207.2016282	C13(13C)2H24	C15H24	Farnesene	0.04
**5**	209.2086702	C11(13C)4H24	C15H24	Farnesene	0.12
**6**	153.1458941	C7(13C)4H16	C11H16		0.02
**6**	152.1423731	C8(13C)3H16	C11H16		0.02
**6**	151.1393001	C9(13C)2H16	C11H16		0.02
**7**	124.1114331	C6(13C)3H12	C9H12		0.01
**7**	123.1079121	C7(13C)2H12	C9H12		0.01
**8**	96.08900509	C6(13C)1H10	C7H10		0.01
**8**	97.09207809	C5(13C)2H10	C7H10		0.01
**9**	82.07331107	C5(13C)1H8	C6H8		0.01
**9**	83.07683208	C4(13C)2H8	C6H8		0.01
**10**	74.0957671	C2(13C)3H10	C5H10		0.01

**10**	73.09224609	C3(13C)2H10	C5H10		0.05
**10**	72.08872509	C4(13C)1H10	C5H10		0.05
**11**	148.1323211	C5(13C)3H16O2	C8H16O2	Octanoic Acid	0.03
**11**	147.1288001	C6(13C)2H16O2	C8H16O2	Octanoic Acid	0.04
**11**	146.1257271	C7(13C)1H16O2	C8H16O2	Octanoic Acid	0.03
**12**	205.1977222	C8(13C)4H24O2	C12H24O2	Ethyl decanoate	0.03
**12**	206.2012432	C7(13C)5H24O2	C12H24O2	Ethyl decanoate	0.01
**12**	204.1950972	C9(13C)3H24O2	C12H24O2	Ethyl decanoate	0.05
**12**	203.1915762	C10(13C)2H24O2	C12H24O2	Ethyl decanoate	0.04
**12**	202.1880552	C11(13C)1H24O2	C12H24O2	Ethyl decanoate	0.02
**13**	95.0975801	C2(13C)2H12O2	C4H12O2	Ethanol	0.39
**13**	93.09098609	C4H12O2	C4H12O2	Ethanol	0.37
**13**	94.09450709	C3(13C)1H12O2	C4H12O2	Ethanol	0.76
**14**	77.08706609	C2(13C)2H10O	C4H10O		0.07
**14**	76.08399308	C3(13C)1H10O	C4H10O		0.14
**14**	75.08047208	C4H10O	C4H10O		0.07
**15**	71.07669208	C3(13C)2H8	C5H8		0.01
**15**	70.07317107	C4(13C)1H8	C5H8		0.01
**16**	101.0595701	C5H8O2	C5H8O2		0.02
**16**	103.0666121	C3(13C)2H8O2	C5H8O2		0.03
**16**	102.0630911	C4(13C)1H8O2	C5H8O2		0.06
**17**	120.0736051	C4(13C)1H10O3	C5H10O3		0.01
**18**	90.06295106	C3(13C)1H8O2	C4H8O2		0.02
**19**	106.0731431	C7(13C)1H8	C8H8		-0.02
**19**	105.0700701	C8H8	C8H8		-0.01
**20**	62.03201103	C1(13C)1H4O2	C2H4O2	Acetic acid	-0.04
**20**	61.02849003	C2H4O2	C2H4O2	Acetic acid	-0.04

Table S4. Compounds contributing to PC2 (*i.e.*, absolute value loading > 0.01)

#	*m/z*	molecular formula (^{12}C & ^{13}C)	Unique molecular formula	ID	Loading value

**1**	227.0386	C9(13C)1H3N5O2	C10H3N5O2		0.13
**1**	226.0356	C10H3N5O2	C10H3N5O2		0.12
**1**	228.0422	C8(13C)2H3N5O2	C10H3N5O2		0.02
**2**	228.0314	C9(13C)2H3N3O3	C11H3N3O3		0.01
**3**	192.0480	C8(13C)1H6N2O3	C9H6N2O3		0.07
**4**	172.1326	C7(13C)3H16O2	C10H16O2	Geranic acid	0.03
**4**	170.1256	C9(13C)1H16O2	C10H16O2	Geranic acid	0.03
**4**	171.1291	C8(13C)2H16O2	C10H16O2	Geranic acid	0.05
**5**	102.0268	C3(13C)1H4O3	C4H4O3		0.01
**6**	192.1676	C9(13C)1H22O3	C10H22O3		0.02
**6**	191.1641	C10H22O3	C10H22O3		0.17
**7**	201.1120	C10H16O4	C10H16O4		0.02
**8**	160.0684	C6(13C)1H10O4	C7H10O4		0.03
**8**	159.0653	C7H10O4	C7H10O4		0.34
**9**	236.1129	C7(13C)3H16O6	C10H16O6		0.03
**10**	113.0508	C4(13C)2H6O2	C6H6O2		0.01
**11**	100.0475	C4(13C)1H6O2	C5H6O2		0.05
**11**	101.0506	C3(13C)2H6O2	C5H6O2		0.04
**12**	104.0424	C3(13C)1H6O3	C4H6O3		0.01
**13**	76.0392	C2H5NO2	C2H5NO2		0.02
**14**	188.1276	C7(13C)3H16O3	C10H16O3		0.04
**14**	187.1240	C8(13C)2H16O3	C10H16O3		0.04
**14**	186.1205	C9(13C)1H16O3	C10H16O3		0.02
**15**	169.1135	C8(13C)2H14O2	C10H14O2		0.01
**16**	144.0734	C6(13C)1H10O3	C7H10O3		0.03
**16**	145.0770	C5(13C)2H10O3	C7H10O3		0.03
**17**	131.0613	C4(13C)2H8O3	C6H8O3		0.03
**17**	130.0577	C5(13C)1H8O3	C6H8O3		0.04
**18**	114.0632	C5(13C)1H8O2	C6H8O2	Sorbic acid	0.06
**18**	115.0663	C4(13C)2H8O2	C6H8O2	Sorbic acid	0.05
**19**	89.0509	C2(13C)2H6O2	C4H6O2		0.01

**19**	88.0474	C3(13C)1H6O2	C4H6O2	0.03
**20**	133.0768	C4(13C)2H10O3	C6H10O3	0.02
**20**	132.0737	C5(13C)1H10O3	C6H10O3	0.03
**21**	129.0820	C5(13C)2H10O2	C7H10O2	0.03
**22**	115.1026	C5(13C)2H12O	C7H12O	0.01
**23**	101.0873	C4(13C)2H10O	C6H10O	0.01
**24**	157.1134	C7(13C)2H14O2	C9H14O2	0.01
**25**	113.0870	C5(13C)2H10O	C7H10O	0.01
**26**	99.0713	C4(13C)2H8O	C6H8O	0.01
**26**	98.0683	C5(13C)1H8O	C6H8O	0.01
**27**	160.0962	C5(13C)3H12O3	C8H12O3	0.02
**27**	159.0927	C6(13C)2H12O3	C8H12O3	0.04
**27**	158.0891	C7(13C)1H12O3	C8H12O3	0.03
**28**	142.0942	C7(13C)1H12O2	C8H12O2	0.04
**28**	144.1012	C5(13C)3H12O2	C8H12O2	0.03
**28**	143.0977	C6(13C)2H12O2	C8H12O2	0.06
**29**	147.1198	C4(13C)4H14O2	C8H14O2	0.02
**29**	144.1102	C7(13C)1H14O2	C8H14O2	0.07
**29**	146.1168	C5(13C)3H14O2	C8H14O2	0.06
**29**	145.1132	C6(13C)2H14O2	C8H14O2	0.10
**30**	162.1117	C5(13C)3H14O3	C8H14O3	0.01
**30**	161.1082	C6(13C)2H14O3	C8H14O3	0.02
**30**	160.1051	C7(13C)1H14O3	C8H14O3	0.02
**31**	127.1027	C6(13C)2H12O	C8H12O	0.06
**31**	128.1058	C5(13C)3H12O	C8H12O	0.02
**32**	127.0664	C5(13C)2H8O2	C7H8O2	0.01
**33**	157.0771	C6(13C)2H10O3	C8H10O3	0.01
**34**	141.0821	C6(13C)2H10O2	C8H10O2	0.01
**34**	140.0786	C7(13C)1H10O2	C8H10O2	0.01
**35**	125.0872	C6(13C)2H10O	C8H10O	0.01
**36**	143.0614	C5(13C)2H8O3	C7H8O3	0.01

**36**	142.0579	C6(13C)1H8O3	C7H8O3		0.01
**37**	116.0425	C4(13C)1H6O3	C5H6O3		0.04
**37**	117.0456	C3(13C)2H6O3	C5H6O3		0.01
**38**	178.1703	C5(13C)5H20O2	C10H20O2	Ethyl octanoate	0.01
**38**	177.1668	C6(13C)4H20O2	C10H20O2	Ethyl octanoate	0.06
**38**	176.1637	C7(13C)3H20O2	C10H20O2	Ethyl octanoate	0.16
**38**	175.1606	C8(13C)2H20O2	C10H20O2	Ethyl octanoate	0.20
**38**	174.1571	C9(13C)1H20O2	C10H20O2	Ethyl octanoate	0.12
**39**	126.1270	C6(13C)3H14	C9H14		0.01
**39**	125.1235	C7(13C)2H14	C9H14		0.01
**40**	112.1113	C5(13C)3H12	C8H12		0.01
**40**	111.1078	C6(13C)2H12	C8H12		0.01
**41**	212.2188	C8(13C)7H24	C15H24	Farnesene	0.01
**41**	211.2153	C9(13C)6H24	C15H24	Farnesene	0.04
**41**	210.2122	C10(13C)5H24	C15H24	Farnesene	0.08
**41**	208.2051	C12(13C)3H24	C15H24	Farnesene	0.09
**41**	207.2016	C13(13C)2H24	C15H24	Farnesene	0.04
**41**	209.2087	C11(13C)4H24	C15H24	Farnesene	0.11
**42**	124.1114	C6(13C)3H12	C9H12		0.01
**42**	123.1079	C7(13C)2H12	C9H12		0.01
**43**	152.1424	C8(13C)3H16	C11H16		0.02
**43**	151.1393	C9(13C)2H16	C11H16		0.01
**44**	96.0890	C6(13C)1H10	C7H10		0.01
**44**	98.0956	C4(13C)3H10	C7H10		0.01
**44**	97.0921	C5(13C)2H10	C7H10		0.02
**45**	74.0958	C2(13C)3H10	C5H10		0.03
**45**	73.0922	C3(13C)2H10	C5H10		0.13
**45**	72.0887	C4(13C)1H10	C5H10		0.15
**46**	149.1354	C4(13C)4H16O2	C8H16O2	Octanoic acid	0.01
**46**	148.1323	C5(13C)3H16O2	C8H16O2	Octanoic acid	0.07
**46**	147.1288	C6(13C)2H16O2	C8H16O2	Octanoic acid	0.13

**46**	146.1257	C7(13C)1H16O2	C8H16O2	Octanoic acid	0.11
**46**	145.1222	C8H16O2	C8H16O2	Octanoic acid	0.03
**47**	139.1302	C6(13C)4H14	C10H14		0.01
**47**	138.1267	C7(13C)3H14	C10H14		0.02
**47**	137.1236	C8(13C)2H14	C10H14		0.02
**48**	83.0768	C4(13C)2H8	C6H8		0.02
**49**	140.1427	C7(13C)3H16	C10H16		0.01
**50**	108.0887	C7(13C)1H10	C8H10		-0.02
**51**	59.0766	C2(13C)2H8	C4H8		0.01
**51**	58.0735	C3(13C)1H8	C4H8		0.02
**52**	229.2347	C11(13C)4H28O	C15H28O		-0.01
**53**	204.1951	C9(13C)3H24O2	C12H24O2	Ethyl decanoate	0.04
**53**	203.1916	C10(13C)2H24O2	C12H24O2	Ethyl decanoate	0.04
**53**	202.1881	C11(13C)1H24O2	C12H24O2	Ethyl decanoate	0.01
**54**	95.0976	C2(13C)2H12O2	C4H12O2		-0.14
**54**	94.0945	C3(13C)1H12O2	C4H12O2		-0.14
**55**	77.0871	C2(13C)2H10O	C4H10O		-0.02
**55**	76.0840	C3(13C)1H10O	C4H10O		-0.02
**56**	371.1017	C20(13C)3H13NO4	C23H13NO4		0.01
**57**	229.0345	C9H8O7	C9H8O7		0.01
**58**	103.0751	C5H10O2	C5H10O2		0.03
**58**	105.0822	C3(13C)2H10O2	C5H10O2		0.04
**58**	104.0786	C4(13C)1H10O2	C5H10O2		0.07
**59**	133.1136	C5(13C)2H14O2	C7H14O2		0.01
**60**	118.0581	C4(13C)1H8O3	C5H8O3		0.01
**61**	120.1009	C3(13C)3H12O2	C6H12O2	Hexanoic acid	0.01
**61**	119.0979	C4(13C)2H12O2	C6H12O2	Hexanoic acid	0.04
**61**	118.0943	C5(13C)1H12O2	C6H12O2	Hexanoic acid	0.05
**62**	87.0717	C3(13C)2H8O	C5H8O		0.02
**62**	86.0681	C4(13C)1H8O	C5H8O		0.03
**63**	190.1431	C7(13C)3H18O3	C10H18O3		0.02

**63**	189.1396	C8(13C)2H18O3	C10H18O3		0.02
**63**	188.1361	C9(13C)1H18O3	C10H18O3		0.01
**64**	176.1547	C5(13C)5H18O2	C10H18O2		0.01
**64**	175.1512	C6(13C)4H18O2	C10H18O2		0.05
**64**	172.1411	C9(13C)1H18O2	C10H18O2		0.06
**64**	173.1446	C8(13C)2H18O2	C10H18O2		0.12
**64**	174.1482	C7(13C)3H18O2	C10H18O2		0.11
**65**	157.1407	C6(13C)4H16O	C10H16O	Citral	0.03
**65**	156.1376	C7(13C)3H16O	C10H16O	Citral	0.07
**65**	155.1341	C8(13C)2H16O	C10H16O	Citral	0.08
**66**	104.0697	C2(13C)3H8O2	C5H8O2		0.02
**66**	101.0596	C5H8O2	C5H8O2		0.20
**66**	103.0666	C3(13C)2H8O2	C5H8O2		0.22
**66**	102.0631	C4(13C)1H8O2	C5H8O2		0.47
**67**	120.0736	C4(13C)1H10O3	C5H10O3		0.06
**67**	121.0771	C3(13C)2H10O3	C5H10O3		0.05
**68**	202.1795	C9(13C)3H22O2	C12H22O2		0.02
**68**	201.1760	C10(13C)2H22O2	C12H22O2		0.01
**69**	77.0597	C3H8O2	C3H8O2		-0.02
**70**	74.0680	C3(13C)1H8O	C4H8O		0.01
**71**	160.1325	C6(13C)3H16O2	C9H16O2		0.01
**71**	159.1289	C7(13C)2H16O2	C9H16O2		0.01
**72**	153.1186	C8(13C)2H14O	C10H14O		0.02
**73**	117.0819	C4(13C)2H10O2	C6H10O2		0.03
**73**	116.0788	C5(13C)1H10O2	C6H10O2		0.03
**74**	130.0945	C6(13C)1H12O2	C7H12O2		0.01
**74**	131.0976	C5(13C)2H12O2	C7H12O2		0.02
**75**	89.0599	C4H8O2	C4H8O2		0.09
**75**	91.0665	C2(13C)2H8O2	C4H8O2		0.06
**75**	90.0630	C3(13C)1H8O2	C4H8O2		0.19
**76**	73.0560	C2(13C)2H6O	C4H6O		0.01

**76**	72.0524	C3(13C)1H6O	C4H6O	0.03
**77**	107.0767	C6(13C)2H8	C8H8	0.12
**77**	106.0731	C7(13C)1H8	C8H8	0.21
**77**	105.0701	C8H8	C8H8	0.09
**78**	62.0320	C1(13C)1H4O2	C2H4O2	-0.02
**78**	61.0285	C2H4O2	C2H4O2	0.04
**79**	84.0889	C5(13C)1H10	C6H10	-0.02
**80**	98.1046	C6(13C)1H12	C7H12	-0.01
**81**	58.0367	C2(13C)1H4O	C3H4O	-0.01
**82**	147.1566	C5(13C)4H18O	C9H18O	-0.01
**82**	146.1531	C6(13C)3H18O	C9H18O	-0.02
**82**	145.1495	C7(13C)2H18O	C9H18O	-0.02
**82**	144.1465	C8(13C)1H18O	C9H18O	-0.02
**83**	172.1778	C10(13C)1H22O	C11H22O	-0.01
**83**	175.1880	C7(13C)4H22O	C11H22O	-0.02
**83**	174.1844	C8(13C)3H22O	C11H22O	-0.02
**83**	173.1809	C9(13C)2H22O	C11H22O	-0.02
**84**	203.2194	C9(13C)4H26O	C13H26O	-0.02
**84**	202.2154	C10(13C)3H26O	C13H26O	-0.02
**85**	118.1217	C4(13C)3H14O	C7H14O	-0.02
**85**	117.1186	C5(13C)2H14O	C7H14O	-0.02
**86**	233.2569	C9(13C)6H30O	C15H30O	-0.01
**86**	229.2437	C13(13C)2H30O	C15H30O	-0.01
**86**	232.2538	C10(13C)5H30O	C15H30O	-0.03
**86**	231.2503	C11(13C)4H30O	C15H30O	-0.03
**86**	230.2472	C12(13C)3H30O	C15H30O	-0.03

Table S5. Compounds detected in negative ion mode during yeast growth in $^{13}C_1$-glucose. All the analytes were detected in $[M-H]^-$ form.

#	*m/z*	molecular formula (^{12}C & ^{13}C)	Unique molecular formula	RDBE	Mass error (ppm)	ID
**1**	58.03793	C2(13C)1H6O	C3H6O	1	-0.23	

**2**	59.01383	C2H4O2	C2H4O2	1	-0.38	Acetic acid
**2**	60.0172	C1(13C)1H4O2	C2H4O2	1	-0.13	Acetic acid
**2**	61.02057	(13C)2H4O2	C2H4O2	1	0.11	Acetic acid
**3**	73.02951	C3H6O2	C3H6O2	1	0.09	Propionic acid
**3**	74.03288	C2(13C)1H6O2	C3H6O2	1	0.29	Propionic acid
**4**	86.0693	C4(13C)1H10O	C5H10O	1	0.65	
**4**	87.07252	C3(13C)2H10O	C5H10O	1	-0.89	
**5**	87.0452	C4H8O2	C4H8O2	1	0.53	Butanoic acid
**5**	88.04857	C3(13C)1H8O2	C4H8O2	1	0.70	Butanoic acid
**5**	89.05194	C2(13C)2H8O2	C4H8O2	1	0.85	Butanoic acid
**5**	90.05516	C1(13C)3H8O2	C4H8O2	1	-0.64	Butanoic acid
**6**	104.0708	C2(13C)3H10O2	C5H10O2	1	-0.65	Pentanoic acid
**7**	113.0973	C7H14O	C7H14O	1	0.98	Heptanoic acid
**7**	114.1005	C6(13C)1H14O	C7H14O	1	-0.38	Heptanoic acid
**7**	115.1039	C5(13C)2H14O	C7H14O	1	0.01	Heptanoic acid
**7**	116.1073	C4(13C)3H14O	C7H14O	1	0.40	Heptanoic acid
**8**	115.0766	C6H12O2	C6H12O2	1	1.26	Hexanoic acid
**8**	116.0799	C5(13C)1H12O2	C6H12O2	1	0.78	Hexanoic acid
**8**	117.0832	C4(13C)2H12O2	C6H12O2	1	0.31	Hexanoic acid
**8**	118.0865	C3(13C)3H12O2	C6H12O2	1	-0.15	Hexanoic acid
**9**	119.0349	C4H8O4	C4H8O4	1	-0.69	
**9**	120.0383	C3(13C)1H8O4	C4H8O4	1	-0.31	
**9**	121.0417	C2(13C)2H8O4	C4H8O4	1	0.06	
**10**	134.054	C4(13C)1H10O4	C5H10O4	1	0.09	
**10**	135.0574	C3(13C)2H10O4	C5H10O4	1	0.42	
**11**	142.1317	C8(13C)1H18O	C9H18O	1	-1.00	
**11**	143.1354	C7(13C)2H18O	C9H18O	1	1.40	
**11**	144.1388	C6(13C)3H18O	C9H18O	1	1.70	
**11**	145.1426	C5(13C)4H18O	C9H18O	1	4.73	
**12**	143.1076	C8H16O2	C8H16O2	1	-1.06	Octanoic acid
**12**	144.1112	C7(13C)1H16O2	C8H16O2	1	0.63	Octanoic acid

**12**	145.1144	C6(13C)2H16O2	C8H16O2	1	-0.43	Octanoic acid
**12**	146.1179	C5(13C)3H16O2	C8H16O2	1	0.56	Octanoic acid
**12**	147.1211	C4(13C)4H16O2	C8H16O2	1	-0.50	Octanoic acid
**12**	148.1243	C3(13C)5H16O2	C8H16O2	1	-1.53	Octanoic acid
**13**	147.0665	C6H12O4	C6H12O4	1	1.47	
**13**	148.0695	C5(13C)1H12O4	C6H12O4	1	-0.92	
**14**	162.0854	C6(13C)1H14O4	C7H14O4	1	0.69	
**15**	172.1427	C9(13C)1H20O2	C10H20O2	1	1.68	Decanoic acid
**15**	173.1459	C8(13C)2H20O2	C10H20O2	1	0.78	Decanoic acid
**15**	174.149	C7(13C)3H20O2	C10H20O2	1	-0.68	Decanoic acid
**15**	175.1522	C6(13C)4H20O2	C10H20O2	1	-1.55	Decanoic acid
**15**	176.1556	C5(13C)5H20O2	C10H20O2	1	-1.29	Decanoic acid
**16**	175.0971	C8H16O4	C8H16O4	1	-2.74	
**16**	176.1006	C7(13C)1H16O4	C8H16O4	1	-1.91	
**16**	177.1043	C6(13C)2H16O4	C8H16O4	1	0.04	
**17**	188.1373	C9(13C)1H20O3	C10H20O3	1	-0.12	
**17**	189.1408	C8(13C)2H20O3	C10H20O3	1	0.64	
**17**	190.1441	C7(13C)3H20O3	C10H20O3	1	0.35	
**17**	191.1474	C6(13C)4H20O3	C10H20O3	1	0.06	
**18**	189.1134	C9H18O4	C9H18O4	1	0.88	
**18**	190.1164	C8(13C)1H18O4	C9H18O4	1	-0.98	
**18**	191.1203	C7(13C)2H18O4	C9H18O4	1	1.86	
**18**	192.1233	C6(13C)3H18O4	C9H18O4	1	0.01	
**18**	193.1266	C5(13C)4H18O4	C9H18O4	1	-0.27	
**19**	200.1734	C11(13C)1H24O2	C12H24O2	1	-1.53	Dodecanoic acid
**19**	201.1773	C10(13C)2H24O2	C12H24O2	1	1.17	Dodecanoic acid
**19**	202.1803	C9(13C)3H24O2	C12H24O2	1	-0.58	Dodecanoic acid
**19**	203.184	C8(13C)4H24O2	C12H24O2	1	1.11	Dodecanoic acid
**19**	204.1868	C7(13C)5H24O2	C12H24O2	1	-1.60	Dodecanoic acid
**19**	205.1908	C6(13C)6H24O2	C12H24O2	1	1.53	Dodecanoic acid
**20**	206.1392	C7(13C)3H20O4	C10H20O4	1	1.22	

**21**	218.1477	C10(13C)1H22O4	C11H22O4	1	-0.86	
**21**	219.1513	C9(13C)2H22O4	C11H22O4	1	0.26	
**21**	220.1547	C8(13C)3H22O4	C11H22O4	1	0.46	
**22**	229.2086	C12(13C)2H28O2	C14H28O2	1	1.03	Tetradecanoic acid
**22**	230.212	C11(13C)3H28O2	C14H28O2	1	1.22	Tetradecanoic acid
**22**	232.2186	C9(13C)5H28O2	C14H28O2	1	0.73	Tetradecanoic acid
**23**	234.1693	C9(13C)3H24O4	C12H24O4	1	-4.03	
**24**	246.1795	C12(13C)1H26O4	C13H26O4	1	1.26	Tridecanoic acid
**24**	247.1828	C11(13C)2H26O4	C13H26O4	1	1.04	Tridecanoic acid
**24**	248.1861	C10(13C)3H26O4	C13H26O4	1	0.81	Tridecanoic acid
**24**	249.189	C9(13C)4H26O4	C13H26O4	1	-1.01	Tridecanoic acid
**25**	101.0519	C3(13C)2H8O2	C5H8O2	2	0.36	
**26**	103.0312	C2(13C)2H6O3	C4H6O3	2	0.70	
**27**	114.0641	C5(13C)1H10O2	C6H10O2	2	-0.51	
**28**	119.026	C2(13C)2H6O4	C4H6O4	2	-0.35	
**29**	131.0624	C4(13C)2H10O3	C6H10O3	2	-0.21	
**29**	132.0656	C3(13C)3H10O3	C6H10O3	2	-1.37	
**30**	142.0956	C7(13C)1H14O2	C8H14O2	2	0.99	
**30**	143.0991	C6(13C)2H14O2	C8H14O2	2	1.99	
**30**	144.1022	C5(13C)3H14O2	C8H14O2	2	0.22	
**31**	145.0781	C5(13C)2H12O3	C7H12O3	2	0.15	
**31**	146.0818	C4(13C)3H12O3	C7H12O3	2	2.50	
**32**	158.0904	C7(13C)1H14O3	C8H14O3	2	0.17	
**32**	159.0938	C6(13C)2H14O3	C8H14O3	2	0.45	
**32**	160.0968	C5(13C)3H14O3	C8H14O3	2	-1.76	
**32**	161.1005	C4(13C)4H14O3	C8H14O3	2	0.38	
**33**	169.1236	C10H18O2	C10H18O2	2	1.16	
**33**	170.1268	C9(13C)1H18O2	C10H18O2	2	0.24	
**33**	171.1303	C8(13C)2H18O2	C10H18O2	2	1.08	
**33**	172.1334	C7(13C)3H18O2	C10H18O2	2	-0.40	
**33**	173.1374	C6(13C)4H18O2	C10H18O2	2	3.31	

**34**	175.0887	C6(13C)2H14O4	C8H14O4	2	0.33
**34**	176.0919	C5(13C)3H14O4	C8H14O4	2	-0.55
**35**	186.1218	C9(13C)1H18O3	C10H18O3	2	0.68
**35**	187.1251	C8(13C)2H18O3	C10H18O3	2	0.38
**35**	188.1282	C7(13C)3H18O3	C10H18O3	2	-0.97
**35**	189.132	C6(13C)4H18O3	C10H18O3	2	1.38
**36**	216.1596	C9(13C)3H22O3	C12H22O3	2	-0.38
**36**	217.1628	C8(13C)4H22O3	C12H22O3	2	-1.09
**37**	221.067	C8H14O7	C8H14O7	2	1.46
**37**	222.0699	C7(13C)1H14O7	C8H14O7	2	-0.59
**38**	177.1088	(13C)6H12N4O2	C6H12N4O2	3	-0.44
**39**	206.1426	C1(13C)7H16N4O2	C8H16N4O2	3	-4.51
**39**	207.1466	(13C)8H16N4O2	C8H16N4O2	3	-1.39
**40**	174.0897	C1(13C)5H10N4O2	C6H10N4O2	4	-0.99
**41**	203.125	C2(13C)6H14N4O2	C8H14N4O2	4	2.31
**41**	204.1281	C1(13C)7H14N4O2	C8H14N4O2	4	1.05
**41**	205.1312	(13C)8H14N4O2	C8H14N4O2	4	-0.19
**42**	231.2154	C3(13C)12H24O	C15H24O	4	-1.29
**43**	215.1567	C3(13C)10H18O2	C13H18O2	5	-1.17
